# Control efficiency and Huanglongbing resistance-related clues mediated by novel *Hermetia illucens frass* formulation

**DOI:** 10.3389/fmicb.2026.1896630

**Published:** 2026-08-04

**Authors:** Zhi-Cheng Ding, Yang Liu, Shao-Ran Zhang, Yue-Hua Yang, Jia-Lin Jiang, Ling Jiang

**Affiliations:** 1 National Key Laboratory of Germplasm Innovation and Utilization of Fruit and Vegetable Horticultural Crops, College of Horticulture and Forestry, Huazhong Agricultural University, Wuhan, China; 2 National Key Laboratory of Agricultural Microbial, Huazhong Agricultural University, Wuhan, China

**Keywords:** antimicrobial peptides (AMPs), *Hermetia illucens frass* (HIF), Huanglongbing (HLB), microbial fertilizer, omics, phytohormone

## Abstract

The preventive and curative management of citrus Huanglongbing (HLB) remains a major global challenge in citrus production. As an emerging microbial fertilizer rich in antimicrobial peptide mixtures, *Hermetia illucens frass* (HIF) exhibits promising antibacterial potential against HLB-associated pathogens. In this study, we evaluated the suppressive effects of HIF on *Candidatus* Liberibacter asiaticus (*C*Las) infection and explored the underlying physiological, metabolic, and multi-omic regulatory mechanisms in *C*Las-infected citrus nursery trees grown in pots. Quantitative real-time PCR analysis showed that 20 and 25 consecutive soil applications of HIF significantly reduced *C*Las titers by 52.61% and 61.85%, respectively, and citrus leaves with typical chlorotic and mottled symptoms gradually recovered normal green coloration after 20 rounds of treatment. Phytohormone profiling indicated that the endogenous contents of auxin, cytokinin, and salicylic acid were significantly upregulated following HIF application. Non-targeted metabolomics further revealed that HIF treatment markedly increased the accumulation of 375 and 724 differential metabolites in citrus tissues, including ketones, aldehydes, terpenoids, flavonoids, alkaloids, coumarins, steroids, and polyphenols. Transcriptomic and metabolomic analyses identified 9 significantly upregulated KEGG pathways in leaves and 26 in roots after HIF treatment. Integrated multi-omic comparisons yielded 15 co-upregulated pathways from transcriptome–metabolome pairing, 4 from transcriptome-proteome pairing, and 1 from proteome-metabolome pairing. Notably, α-linolenic acid metabolism was consistently activated across transcriptomic, proteomic, and metabolomic datasets, representing a core conserved signaling pathway responding to HIF treatment. Microbial community analysis characterized the top 10 dominant bacterial genera in both HIF material and HIF-treated citrus tissues. Furthermore, HIF contained abundant antimicrobial secondary metabolites, such as lipids, benzenoids, polyketides, and phenylpropanoids. HPLC detection confirmed the presence of the lipopeptides surfactin and iturin, and metagenomic alignment predicted a total of 467 antimicrobial peptides classified as attacin-like, defensin-like, and cecropin-like peptides. Collectively, these phenotypic, physiological, and multi-omic results provide multi-layered validation clues for research on utilizing HLF to combat Huanglongbing.

## Highlights

- Microbial fertilizer formulation *Hermetia illucens frass* (HIF) significantly reduced the titer of *C*las-infected citrus trees followed both 20 and 25 applications.
- HIF treatment promoted the growth of pot-nursery plants and helped *C*las-infected plants regain vitality.
- The contents of salicylic acid (SA) were significantly upregulated after HIF treatment in citrus.
- Non-targeted metabolomics analysis revealed increased accumulation of lipids, flavonoids, terpenoids and other defensive relative metabolites in citrus after HIF treatment.
- Having antibacterial properties lipopeptides (Surfactin and Iturin) and antimicrobial peptides (AMP) was found existed in HIF.
- Multiple high-abundance dominant bacterial genera were detected in HIF, involving stress tolerance, strengthen the degradation of polysaccharides etc., and enhance the nitrogen cycle and plant growth-promoting trait.
- Co-upregulated transcriptomic, proteomic and metabolomic pathways after HIF treatment reveal clues to verify candidate molecules for HLB resistance.

## 1 Introduction

Citrus Huanglongbing (HLB) is a devastating bacterial disease that seriously threatens the citrus production in nearly 50 countries, especially in US Florida and Brazil (Ghosh et al., 2023, Wong, 2025). HLB is caused by *Candidatus Liberibacter* spp. (*C*Las) (Jagoueix et al., 1994; Teixeira et al., 2005). In CLas-infected tree, phloem tissue necrosis and sieve tube blockage disrupt the translocation of photosynthates (Etxeberria et al., 2009). *C*Las infection also induces root collapse associated with microbial dysbiosis (Gardner et al., 2020), severely affecting both yield and fruit quality (Ponce et al., 2018). Culturing the HLB pathogen *in vitro* remains highly challenging, significantly impeding studies of pathogenic mechanisms and the development of control strategies. In recent years, researchers have proposed and developed numerous innovative strategies, including the screening of disease-resistant germplasm resources (Bao et al., 2023) antibacterial agents to eliminate *C*Las disease (Pagliai et al., 2014), or *Bacillus* (Li et al., 2022; Asad et al., 2021), and the application of antimicrobial peptide SAMP (Huang et al., 2021), APP-14 peptide (Zhao et al., 2025), however, the stability and durability effectiveness in the field application scenarios still needs to be verified.

*Hermetia illucens frass* (HIF) microbial fertilizer formulation containing plant nutrients, lipo-peptides and antimicrobial peptides possesses the potential to control of citrus greening disease. The black soldier fly (*Hermetia illucens* L.) is a globally distributed dipteran insect that has been extensively studied. The HIF is composed of feces and organic residues remaining after the digestion and absorption of organic waste as well as abundant intestinal microbial flora from black soldier fly larvae (Cifuentes et al., 2022). Due to exposure to high concentrations of harmful substances during feeding, larvae are subjected to complex environmental pressures that stimulate the production of various antimicrobial cyclic lipopeptides (CLPs), such as surfactin and iturin and antimicrobial peptides (AMPs) (Park et al., 2015; Zhan et al., 2020; Manniello et al., 2021). Some of these peptides (e.g., cecropin) are particularly effective against Gram-negative bacteria (Gimranov et al., 2025; Sharma et al., 2000). In addition, black soldier fly dung serves as an effective soil conditioner, enhancing soil health whereas functioning as an environmentally friendly microbial fertilizer (Yang et al., 2021). During transformation, the dung temperature typically remains between 28 and 35°C, supporting a highly diverse microbial community (Arnone et al., 2022). HIF produced by black soldier fly larvae is a high-quality microbial fertilizer rich in microorganisms and secondary metabolites, providing significant potential for enhancing plant disease resistance.

To provide new strategy for more safety, effectiveness, sustainability citrus HLB control and tree growth improvement, the application effect of HIF microbial fertilizer formulation has an urgent need to explore.

## 2 Materials and methods

### 2.1 Preparation and application of HIF formulation

HIF was provided by Wuhan Kewei Microbial Technology Co., Ltd. This product uses chicken manure as raw material. 6-day-old black soldier fly larvae were inoculated and transformed for 8 days. The black soldier fly larvae were screened out, and the remaining part was the HIF powder. The quality inspection performed by the Microbial Product Quality Monitoring and Testing Center of the Ministry of Agriculture and Rural Affairs indicates that the product contains more than 200 million CFU/g of microbials, with total nutrients (N + P_2_O_5_ + K_2_O) ≥ 10%, organic matter ≥ 40%, and test results that conformed to the NY/T 525-2021 standard. For the HIF treatment group, 20 g/plant was applied to citrus plants grown in plastic pots (diameter 28–32 cm, height 30 cm). The HIF as evenly distributed 10–15 cm away from the main trunk and lightly incorporated into the soil.

qPCR analysis was performed on citrus plants, and *C*Las-infected plants were prepared for subsequent experiments. HIF was applied once every 7 days for 20 and 25 applications.

During the experimental period, all plants received 10 g/plant of compound granular fertilizer (N: P_2_O_5_: K_2_O = 14:16:15, Yichang Fusheng Chemical Co., Ltd.), which was applied to the soil. Trace elements of Ca + Mg ≥ 10%, S ≥ 12%, Zn ≥ 7%, B ≥ 0.06%, Mn ≥ 0.1% from Shandong Woli Biotechnology Group Co., Ltd.) at 0.1 g/plant were dissolved in 500 mL water and sprayed on the leaves in May, July, and December as part of the regular management. Cultivation management was identical for both the HIF treatment group and control groups, except that the control group did not receive HIF.

### 2.2 Determination of citrus morphological and physiological indexes

Plant height was measured from the ground surface to the apex of the plants in 20 HLF soil applications. Crown diameter was calculated as the average of two perpendicular diameters measured along the north–south and east–west directions of the canopy, with five biological replicates. For leaf thickness measurement, 50 mature leaves collected per plant, 10 leaves were stacked and aligned carefully; measurements were taken using an electronic digital caliper while avoiding major veins, and the mean was calculated. Root projected area was determined from original images using ImageJ software. SPAD values and leaf nitrogen content were measured with a TYSND0 plant nutrition analyzer manufactured by Zhejiang Top Instrument Co., Ltd. All statistical analyses were performed using SPSS software, with significance evaluated by independent-sample *t*-test.

### 2.3 Determination of *C*Las before and after HIF treatment

#### 2.3.1 DNA extraction

Two-year-old “*Citrus reticulata cv. Kinokuni*” *plants*, grafted on *Poncirus trifoliata* rootstock, were cultivated under isolation in net greenhouses. Veins samples were collected from plants. For each plant, mixed samples were taken from the third to fifth leaves on four directional branches. Tools were disinfected with 4% (v/v) NaClO. After cutting the leaf veins, tissues were placed in centrifuge tubes, frozen in liquid nitrogen, and stored at –70°C. Midveins (100 mg per sample) were collected for DNA extraction using the CTAB method (Fallah et al., 2017), or DNA extraction using Plant Genomic DNA Kit and purification DNA with adsorption column (Beijing ZOMAMBIO Biottechnology Co., Ltd).

#### 2.3.2 qPCR detection of *C*Las titer

The extracted DNA was analyzed by qPCR with the Corbett RG-6000, Designed and manufactured in Sydney, Australia. The cytochrome oxidase (*COX*) gene served as the internal reference, with primers *COX* + (5’-GTATGCCACGTCGCATTCCAGA-3’) and *COX–* (5’-CCAAAACTGCTAAGGGCATTC-3’) (Li et al., 2006). The target gene was the ribonucleotide reductase (*RNR)* gene of the Asian-type HLB pathogen, with primers *RNR* + (5’-CATGCTCCATGAAGCTACCC-3’) and *RNR–* (5’-GGAGCATTTAACCCCACGAA-3’) (Zheng et al., 2016). For SYBR Green real-time PCR, the reaction mixture refer to 2 × Realtime PCR Super mix (Mei5 Biotechnology Co., Ltd.), or 2 × Universal Sybr Green qPCR Mix (Keep Biotechnologies Co., Ltd). 1 μL of DNA template (∼25 ng), 0.5 μL of each forward and reverse primer (10 μM) in a final volume of 20 μL. The qPCR protocol consisted of pre-denaturation at 95°C for 3 min, denaturation at 95°C for 30 s, annealing at 57°C for 35 s, extension at 72°C for 45 s, and 40 cycles in total, 72°C for 10 min. three biological replicates were set for each sample. DNA from pre-tested healthy “Valencia orange” served as the negative control, and leaf vein DNA from “*Citrus reticulata* “*Shiyue Ju*”” (laboratory-confirmed positive) was used as the positive control.

#### 2.3.3 Preparation of qPCR primer standard curves

PCR amplification products of *COX* and *RNR* genes were ligated with the PcloneZ-NRS vector using the PcloneZ-NRS-OmNI-Kan HC cloning kit. The products were validated and sequenced by agarose gel electrophoresis, then used to generate two standard plasmids for concentration gradient experiments. Standard curves plotting molecule number versus CT value were constructed. The amplification efficiency for each gene was calculated as E = (10^–1/slope^ −1) × 100%, using the slope of the standard curve. The minimum detection limit was determined from the CT value corresponding to the lowest plasmid concentration producing a detectable amplification curve. Efficiency and detection limits for each primer were recorded. Changes in pathogen titer between treatment and control were analyzed for significance using two-proportion z-tests.

### 2.4 Phytohormone determination of leaves and roots in citrus

After the 10th HIF processing, sampling was conducted at 24 h, each sample was ground in liquid nitrogen, and 0.1 g aliquots were added to 1 × mass spectrometry–grade water, three biological replicates. Internal Standard such as,2H4-Indole-3-acetic acid, 2H4-Salicylic acid, 2H4-Salicylic acid etc. used for preparing the equation of the calibration curve. Hormone quantification was performed in accordance with the guidelines of the American Society for Clinical and Laboratory Standards (CLSI) (Wayne, PA: Clinical and Laboratory Standards Institute [CLSI], 2014). An ultra-high performance liquid chromatography coupled to tandem mass spectrometry (UHPLC-MS/MS) system (ExionLC AD UHPLC-QTRAP^®^ 6500 + , AB SCIEX Corp., Boston, MA, United States), with quantification based on internal standard calibration curves. The determination was conducted at Beijing Novogene Technology Co., Ltd.

### 2.5 16 S amplicon analysis of HIF-treated tissue using QIIME2

After 10 treatments, samples were selected from both the HIF treatment and control groups, three biological replicates, Leaves and roots were collected separately, frozen in liquid nitrogen.

Genomic DNA was extracted, and the bacterial 16SV34 region was amplified using primers 341F/806R (5’-CCTAYGGGRBGCASCAG-3’, 5’-GGACTACNNGGGTATCTAA T-3’). The paired-end (PE) sequences generated by Illumina sequencing were assembled, and overlapping regions were merged using Flash 1.2.11. Sequence quality control and filtering were performed with Fastp version 0.23.1 (Bokulich et al., 2018). Effective tags were denoised with Amplicon Sequence Variants (ASV). Subsequently, the obtained valid data were subjected to species annotation and abundance analysis, thereby revealing the composition of the sample species. Carry out further analyses of alpha diversity and beta diversity. The detection was conducted at Beijing Novogene Technology Co., Ltd.

### 2.6 Transcriptomic analysis

#### 2.6.1 Plant materials and sampling

“*Citrus reticulata cv. Kinokuni*” plants were selected from the HIF treatment group and control group. After 10 treatments, leaf samples were collected 12 h after the last treatment. For each plant, the leaves were collected from each of the four cardinal directions (east, west, south, and north) and mixed as one composite sample. Root samples were also prepared as a mixture from roots of the four directions, with four biological replicates per treatment.

#### 2.6.2 RNA extraction and data analysis

Total RNA was extracted using TRIzol reagent. Paired-end (PE) libraries were constructed following the instructions of the ABclonal mRNA-seq Library Preparation Kit (ABclonal, China). Library quality was assessed using an Agilent Bioanalyzer 4150, and sequencing was performed on the NovaSeq 6000 platform with a PE150 read length.

Clean reads were mapped to the Citrus reticulata reference genome using HISAT2. Differential gene expression analysis was performed using the DESeq2 R package (Love et al., 2014). Genes with |log_2_(fold change) | > 1 and adjusted *P*-value (Padj) < 0.05 were defined as significantly differentially expressed genes (DEGs). KEGG pathway enrichment analysis was performed using the clusterProfiler R package, with *P* < 0.05 considered significantly enriched (Kanehisa et al., 2007). All transcriptome sequencing and analyses were performed by APTBIO Biological Co., Ltd.

#### 2.6.3 Quantitative real-time PCR (qRT-PCR) validation

Leaf samples were collected from HIF-treated and control plants at the same time for transcriptomic analysis, with six replicates. Total RNA was extracted from citrus leaves using TAKARA RNAiso Plus reagent following the manufacturer’s protocol. First-strand cDNA was synthesized using a reverse transcription kit from Zhuang Meng Bio-Technology Co., Ltd.

*Actin* was used as the internal reference gene. Citrus callus cDNA served as the negative control. qRT-PCR was performed to determine the relative expression levels of four target genes with primer sequences *cngic1*-F: 5’-TGGCAGTCAAGGTGAAAGA-3’, *cngic1*-R: 5’-GTCCAGCATTGCATTTAAGAA-3’. *mapkk k1*-F: 5’-GGACTGCCTACTCCTCATC-3’, *mapkkk1*-R: 5’-ATA CCAAACAAGCCCTAAA-3’. *paA4g1*-F:5’-CATTGCCGGACTGC TCACC-3’, *paA4g1*-R: 5’-AGAACCCGAAACCCGCTTG-3’. *paA4 g2*-F: 5’-ACCTCGGAGAAATCCACAG-3’, *paA4g2*-R: 5’-CGCTC GTAGTCAGCCTTAT-3’. *Actin*-F: 5’-TGGGACTGGAATGGT GAA-3’, *Actin*-R: 5’-AGTTGCTAACAATGCCATGC-3’. The amplification program was as follows: 94°C for 3 min; 35 cycles of 94°C for 5 s, 57°C for 30 s, and 72°C for 35 s. Relative expression levels were calculated using the 2^–ΔΔCt^ method (Livak and Schmittgen, 2001), and data processing was conducted using Microsoft Excel 2016.

### 2.7 Tandem mass tag (TMT)-based quantitative proteomic analysis

TMT proteomic analysis was performed to identify protein expression changes in leaves and roots under HIF treatment. Samples were collected at the same time points as transcriptomic analysis, with four biological replicates per group (Ross, 2004). Proteins were extracted using SDT lysis buffer (4% (w/v) SDS, 100 mM Tris/HCl pH 7.6, 0.1 M DTT and digested via the filter-aided proteome preparation (FASP) method (Wiśniewski et al., 2009). Peptides were lyophilized, re-dissolved in 40 μL 0.1% formic acid, and quantified at OD280.

Liquid chromatography-tandem mass spectrometry (LC-MS/MS) was performed for peptide identification. Differentially expressed proteins were identified using thresholds of fold change > 1.2 and *P* < 0.05 (Student’s *t*-test). Hierarchical clustering analysis was performed and visualized as a heatmap. KEGG pathway annotation was conducted as previously described (Kanehisa et al., 2012). All proteomic analyses were performed by APTBIO Biotechnology Co., Ltd.

### 2.8 Non-targeted metabolomic analysis

For HIF microbial organic fertilizer preparation, powders were obtained from three independent bags (40 kg per bag), ground in liquid nitrogen (100 mg per sample), with six replicates samples. The Pearson correlation coefficients among pooled QC replicates and principal component analysis (PCA) was performed to evaluate analytical stability (Wen et al., 2017). Metabolite profiling was performed using a Q Exactive HF/X mass spectrometer (Thermo Fisher, Germany) and Vanquish UHPLC system (Thermo Fisher, Germany) equipped with a Hypersil Gold column (100 × 2.1 mm, 1.9 μm; Thermo Fisher, United States). Analysis was carried out by Beijing Novogene Technology Co., Ltd.

For citrus leaves and roots were sampled after 10 rounds of HIF treatment, with six replicates. Samples from four directions were pooled per plant. Non-targeted metabolomic analysis of citrus samples was performed by BMK Technology Co., Ltd.

### 2.9 Determination of lipopeptides in HIF via HPLC

Concentrations of Iturin A and surfactin in HIF were determined by high-performance liquid chromatography (HPLC; Shimadzu) following previously published methods. Sample detection in this experiment was performed at a similar time point as reported in literature Ding et al. (2024).

Determination of Iturin A: Briefly, 0.1 g of HIF powder was dissolved in 1 mL methanol. After centrifugation (4°C, 12,000 r/min, 5 min), the supernatant was collected and filtered through a 0.22 μm organic filter membrane. HPLC analysis was conducted on a SHIMADZU LC20A system equipped with an Agilent TC-C18 column (4.6 × 250 mm, 5 μm). The detection wavelength was 210 nm with the column temperature maintained at 30°C. The mobile phases were 10 mM methanol (A) and pure acetonitrile (B). The initial A/B ratio was 65:35 (V/V), followed by gradient elution (60–93% A, 40–7% B) within 12 min. The flow rate was 1.0 mL/min, and the injection volume was 10 μL.

Determination of surfactin: 0.1 g of HIF powder was dissolved in 1 mL methanol, and the supernatant was prepared as described above. Surfactin was quantified by HPLC at 205 nm and 30°C. The mobile phases and flow rate are same as the above-mentioned inturin detection process. Gradient elution (60%–de% A, 40%–A% B) was performed in 10 min, and the total running time was 20 min.

### 2.10 Sequence alignment of metagenomes for AMP prediction in HIF

For metagenomic sequencing, HIF samples were randomly collected from three bags (40 kg/bag) with three biological replicates. Genomsic DNA was extracted using the PowerSoil DNA Isolation Kit according to the manufacturer’s instructions. Library preparation included DNA fragmentation, end repair and A-tailing, adapter ligation, fragment purification, PCR amplification, and library quality control. Raw reads were filtered using fastp software to obtain clean data. Species annotation at multiple taxonomic levels revealed that the microbial community. Metagenomic sequencing was performed by BMK Biotechnology Co., Ltd. Sequences were aligned against the HIF proteomic reference database using BioEdit for antimicrobial peptide identification (Project code: DZH-61662_blast). BLAST comparisons were also performed against the NCBI proteome database (BioProject: PRJNA683030) as described by Zhang et al. (2022).

## 3 Results

### 3.1 Preparation of specific primer standard curves

Construction map of RNR and COX plasmids showed in Supplementary Figure 1, the amplification curves (Supplementary Figures 2A,B), melting curves (Supplementary Figures 2C,D), and standard curves for the citrus internal reference gene *COX* and the *C*Las *RNR* gene are shown in Supplementary Figures 2E,F, A primer concentration gradient test indicated that, at a primer concentration of 100 pM, the linear regression equation for the *COX* gene was y = –3.3586x + 38.042, with a regression coefficient of *R*^2^ = 0.9983. For the *RNR* gene, the regression equation was y = –3.2484x + 37.727, with *R*^2^ = 0.9907. The amplification efficiency of *COX* + /*COX–* primers reached 98.5%, whereas that of *RNR* + /*RNR–* primers reached 103.2%. These results confirm that *RNR* + /*RNR*– primers provide high repeatability and sensitivity for qPCR detection.

### 3.2 Effect of HIF on the control of Huanglongbing disease

After 20 applications of HIF, the *C*Las pathogen titer decreased by 52.61%, whereas the control group exhibited a 9.03% increased. After 25 applications, the *C*Las titer decreased by 61.85%, whereas the control exhibited a 18.69% reduction. One-way ANOVA revealed a significant overall difference among the three groups (*F* = 3.83, *P* < 0.05). *Post-hoc* LSD tests showed that the titer of CK before processing (5271.68 ± 1693.57) was significantly higher than that of HIF20(2498.30 ± 1399.16) and HIF25(2011.16 ± 1511.21), while no significant difference was detected between HIF 20 and HIF25. Moreover, the control samples at three different time points, no significant difference between any two-control group (Table 1 and Supplementary Figure 3). The relative expression levels (2^–ΔΔCT^) (Livak and Schmittgen, 2001) of the target gene *RNR* were provided in Supplementary Tables 1-1–1-3.

**TABLE 1:** Changes in pathogen titers in citrus veins following HIF treatment.

Sample number	Before processing		After processing 20 times		The pathogen titer decreased (%)	After processing 25 times		The pathogen titer decreased (%)
	CT value ± SD	Average copy number/vein tissue*	CT value ± SD	Average copy number/vein tissue		CT value ± SD	Average copy number/vein tissue	
HIF treatment								
1	25.19 ± 0.03	7234.99	27.03 ± 0.06	1963.38	The pathogen titer decreased 52.61%	29.00 ± 0.03	485.91	The pathogen titer decreased 61.85%
2	25.07 ± 0.04	7877.34	29.03 ± 0.04	475.68		28.34 ± 0.04	775.77	
3	26.20 ± 003	3536.03	26.30 ± 0.03	3294.06		27.04 ± 0.03	1949.52	
4	26.05 ± 0.21	4149.78	26.69 ± 0.01	2498.46		27.60 ± 0.03	1310.79	
5	25.96 ± 0.01	4899.19	25.92 ± 0.04	4312.33		25.91 ± 0.04	4343.01	
6	25.74 ± 0.02	3932.72	26.72 ± 0.04	2445.89		26.34 ± 0.03	3201.97	
	Average	5271.68 ± 1693.57a		2498.30 ± 1399.16b			2011.16 ± 1511.21b	
Control								
CK1	24.93 ± 0.01	8699.17	26.73 ± 0.02	2428.62	The pathogen titer increased 9.03%	24.88 ± 0.04	9013.01	The pathogen titer decreased 18.69%
CK2	24.66 ± 0.03	10534.06	26.32 ± 0.00	3247.69		25.02 ± 0.01	8161.53	
CK3	28.46 ± 0.01	712.51	26.15 ± 0.05	3663.60		25.69 ± 0.01	5075.94	
CK4	25.10 ± 0.01	7711.60	25.08 ± 0.00	7821.70		25.82 ± 0.01	4629.10	
CK5	25.54 ± 0.03	5645.38	24.22 ± 0.03	14389.54		26.43 ± 0.01	3004.08	
CK6	25.63 ± 0.02	5296.48	24.66 ± 0.03	10534.06		27.41 ± 0.02	1499.77	
	Average	6433.19a		7014.20a			5230.57a	

*According to the standard curve Y = −3.2484X + 37.727, CT values were converted to Copy number/Leaf vein tissue. The “different letters” provided in this table denotes the differences between groups reached a significant level (*P* < 0.05).

### 3.3 Effects of HIF on citrus morphological indicators

**FIGURE 1:**
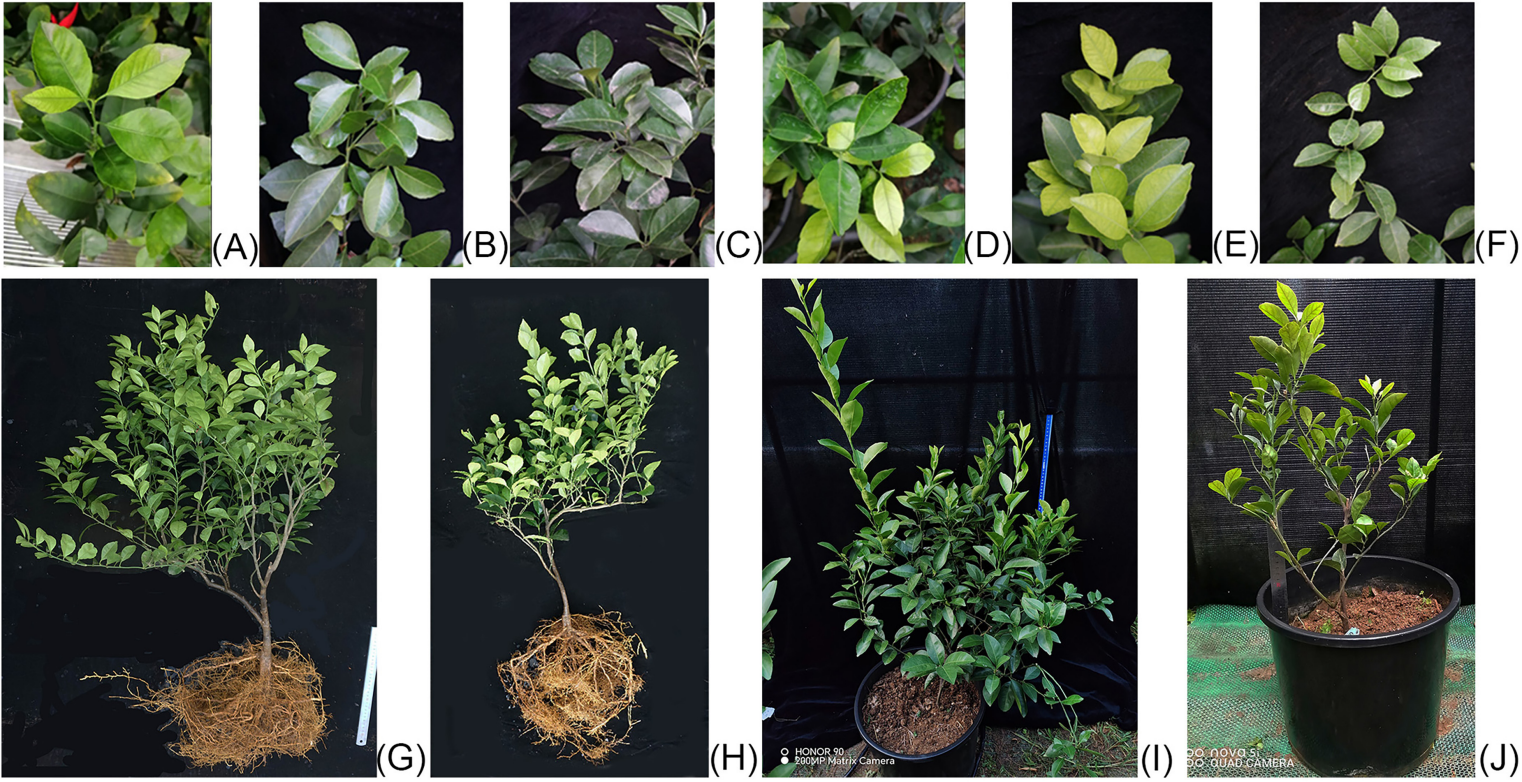
Effect of HIF treatment on citrus morphological characteristics. **(A–C)** HIF-treated plants; (**D–F)** control plants; **(A,D)** photos (chlorotic mottling prior to treatment); **(B)** photos after 12 HIF treatments (leaves green) and **(E)** control (chlorotic leaves); **(C)** photos after 20 HIF treatments (leaves dark green and lustrous); **(F)** control (leaves remained chlorotic); **(G)** plant after 20 HIF treatments (luxuriant leaves, abundant new root growth); **(H)** control plant (poorly developed, browned roots); **(I)** HIF treated plant after 29 months. **(J)** Pre-treatment photo (chlorotic mottling).

Following HIF treatment, citrus leaves and roots showed marked improvements in phenotype. Prior to treatment, the *C*Las-infected plants displayed typical symptoms of yellowing and mottling (Figures 1A,D). Weekly HIF treatments were performed, after 12 treatments, symptoms were alleviated or nearly eliminated (Figure 1B). With 20 treatments, leaves became green, with smooth, waxy surfaces and robust appearance (Figure 1C). The positive control group continued to show yellowed-mottled leaves (Figures 1D–F). Throughout development, plants treated with HIF demonstrated well-developed root systems, vigorous branches, and green foliage (Figure 1G). In contrast, the control group exhibited sparse, browned roots (Figure 1H). The leaves were yellowed and mottled before treatment (Figure 1J), but after 29 months by HIF treatment, plant growth was robust and healthy (Figure 1I), this experiment reflected substantial recovery and growth-promoting effects.

The plant height of citrus in the treatment group was 104.58 ± 13.42 cm, which was significantly higher than that in the control group (84.21 ± 14.39 cm) (*P* = 0.049), observed after HIF processing 20 times. The crown diameter of the treatment group reached 71.24 ± 11.76 cm, which was significantly higher than the control group (55.57 ± 8.07 cm) (*P* = 0.038). The root projected area of the treatment group was 867.69 ± 162.59 cm^2^, which was significantly higher than that of the control group (561.99 ± 188.37 cm^2^) (*P* = 0.025). The results revealed that the leaf thickness of the treatment group (0.27 ± 0.02 mm) was extremely significantly greater than that of the control group (0.25 ± 0.01 mm) (*P* = 0.001) (Table 2).

**TABLE 2:** Promoting effects of HIF treatments on citrus plant and root growth.

Content	Treatment	Control	*P*-value
Plant height (cm)	104.58 ± 13.42a	84.21 ± 14.39b	0.049
Canopy diameter (cm)	71.24 ± 11.76a	55.57 ± 8.07b	0.038
Root projection area(cm^2^)	867.69 ± 162.59a	561.99 ± 188.37b	0.025
Leaf thickness(mm)	0.27 ± 0.02a	0.25 ± 0.01b	<0.01
SPAD value	76.21 ± 2.78a	59.97 ± 6.99b	<0.01
Nitrogen content (mg/g)	23.37 ± 0.83a	18.46 ± 2.30b	<0.01

The “different letters” provided in this table denotes the differences between groups reached a significant level (*P* < 0.05).

The SPAD value of the treatment group was 76.21 ± 2.78, which was extremely significantly higher than that of the control group (59.97 ± 6.99) (*P* = 0.001). The nitrogen content of the treatment group was 23.37 ± 0.83 mg/g, showing an extremely significant increase compared with the control group (18.46 ± 2.30 mg/g) (*P* = 0.001) (Table 2).

### 3.4 HIF treatment induces phytohormonal changes in citrus

**FIGURE 2:**
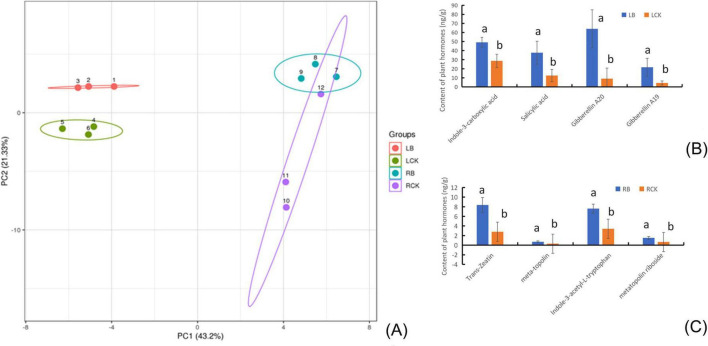
Hormonal content changes following HIF treatment. **(A)** Principal component analysis (PCA) quality control. **(B)** Hormone content changes in leaves. **(C)** Hormone content changes in roots. LB and RB: treatment group, LCK and RCK: control group, L, leaf, R, root. The meaning of “different letters” as provided in this figure is that the differences between groups reached a significant level (*P* < 0.05).

PCA of plant hormone profiles showed that PC1 and PC2 explained 43.2 and 21.33% of the total variance, respectively (Figure 2A), indicating obvious intergroup differences. On-line eXtraction Liquid Chromatography (XIC) chart of hormone standard samples and samples to be tested was showed in Supplementary Figure 4. LC-MS detection revealed that the contents of indole-3-carboxylic acid (ICA), salicylic acid (SA), gibberellin A20 (GA20), and gibberellin A19 (GA19) were significantly upregulated in leaves after HIF treatment, with fold changes of 1.71, 3.03, 7.06, and 5.01 compared to control, respectively (threshold FC > 1.5, *P* < 0.05). The trans-zeatin (tZ), meta-topolin (mT), indole-3-acetyl-L-tryptophan (IAA-Trp), and meta-topolin riboside (mTR) in roots were upregulated by 2.98, 2.37, 2.23, and 2.27-fold (Figures 2B,C and Supplementary Tables 2-1, 2-2). Compared to control, respectively, at 24 h after HIF application. Among these, differentially accumulated hormones were two auxin, four cytokinins, and intermediate and precursor substances involved in the GA1 biosynthesis pathway. These hormones directly or indirectly facilitated cell division, cell elongation and plant growth. Meanwhile, SA could play a role as a key signaling molecule to activate plant immunity and improve disease resistance.

### 3.5 HIF alters bacterial community structure and abundance of citrus

**FIGURE 3:**
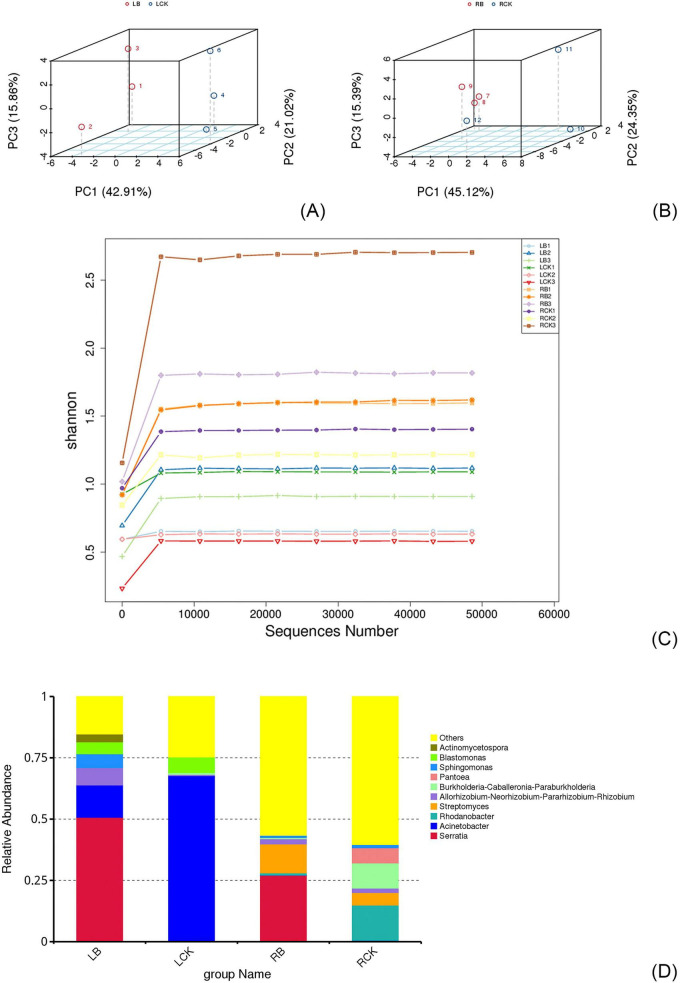
The flora analysis of citrus leaves and roots after HIF treatment. **(A,B)** Principal component analysis-three dimensional (PCA-3D), in leaves and roots. **(C)** Shannon index curves for leaf and root samples. **(D)** Stacked columns represent top 10 genera after HIF treatment.

The leaf and root samples from the HIF treatment experiment were sequenced, with an average of 88,212 effective tags obtained per sample (Supplementary Table 3-1). Principal component analysis-three dimensiona (PCA-3D) revealed that PC1, PC2, and PC3 accounted for 42.91, 21.02, and 15.86% of the total variation in leaf samples, while explaining 45.12, 24.35, and 15.39% of the variation in root samples, respectively (Figures 3A,B). This result indicated distinct differences in microbial community structure between groups and stable clustering within each group. Additionally, the Shannon rarefaction curves of all samples verified adequate sequencing depth (Figure 3C and Supplementary Table 3-2).

Annotation and taxonomic analysis revealed that *Serratia, Acinetobacter, Rhodanobacter, Streptomyces, Allo- rhizobium-Neorhizobium-Pararhizobium-Rhizobium, Burkholderia -Caballeronia-Paraburkholderia, Pantoea, Sphingomonas, Blasto- monas, and Actinomycetospora* comprised the top 10 genera of relatively high abundance. four taxa, such as *Serratia, Allorhizobium_Neorhizobium_Pararhizobium_Rhizobium, Sphin- gomonas*, and *Actinomycetospora* exhibited the higher abundance in leaves following HIF treatment, whereas *Serratia, Streptomyces, Allorhizobium_Neorhizobium_Pararhizobium_Rhizobium were* particularly *more abundant in* HIF- treated *roots* compared with *controls* (Figure 3D and Supplementary Table 3-3).

### 3.6 Transcriptomic analysis of differentially expressed genes and KEGG enrichment

**FIGURE 4:**
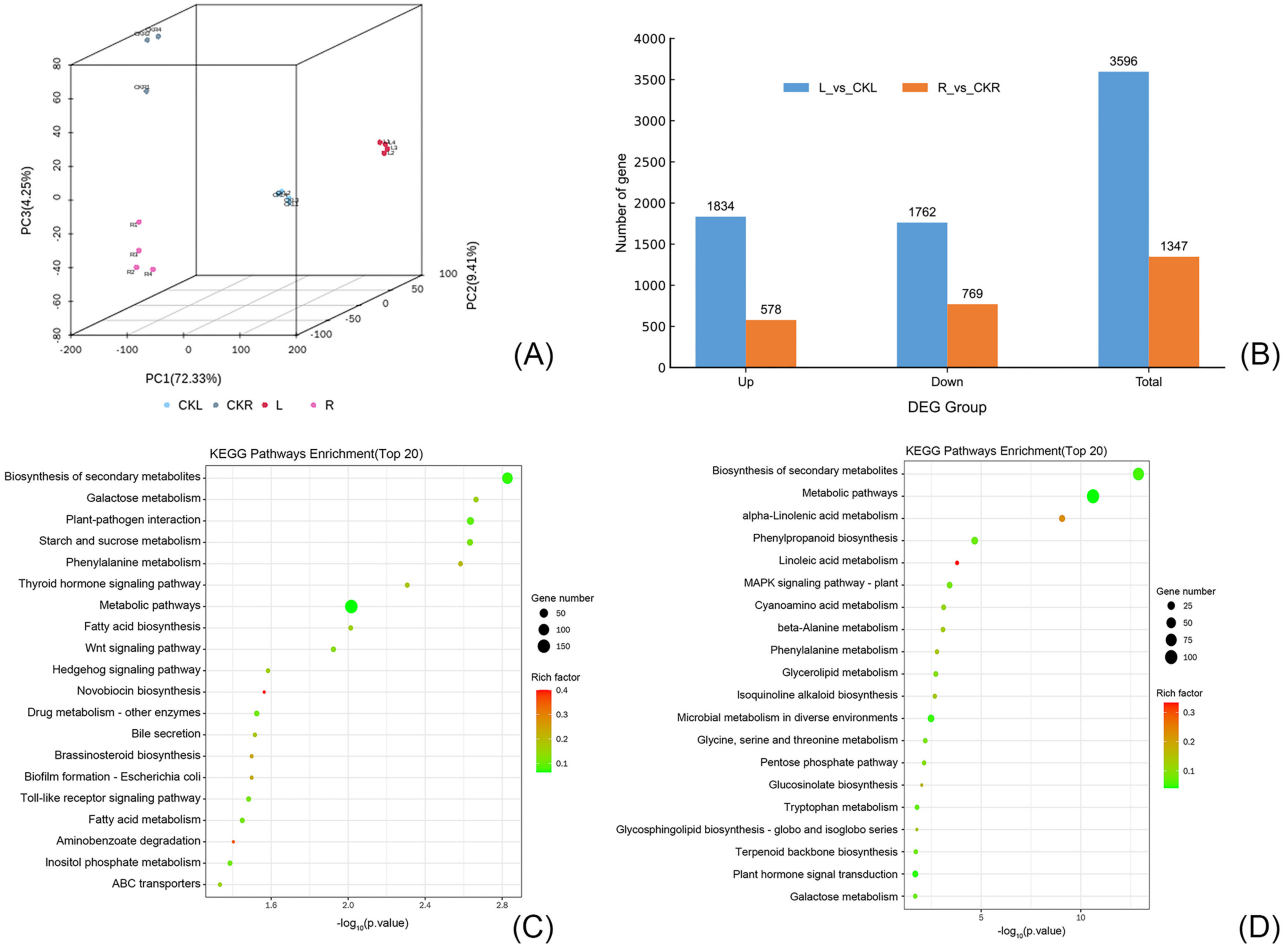
Transcriptome analysis results. **(A)** Principal component analysis-three dimensional (PCA-3D). **(B)** Numbers of differentially expressed genes (DEGs) in leaves and roots. **(C)** Top 20 enriched KEGG pathways in leaves. **(D)** Top 20 enriched KEGG pathways in roots.

Three-dimensional principal component analysis (3D-PCA) was conducted to evaluate sample repeatability and intergroup separation. The results indicated that the contribution rates of PC1, PC2, and PC3 were 72.33, 9.41 and 4.25%, respectively (Figure 4A). Obvious clustering of samples from different groups demonstrated reliable sequencing quality and remarkable biological differences across treatments. In total, 25,984 genes were annotated via transcriptome sequencing in both the HIF treatment and control groups. Differential expression analysis showed that, in leaves, 1,834 significantly upregulated genes and 1,762 downregulated genes were identified in the HIF group relative to the control. In roots, 578 upregulated genes and 769 downregulated genes were detected (Padj < 0.05, |log_2_(FC)| > 1) (Figure 4B and Supplementary Tables 4-1–4-3).

KEGG pathway enrichment analysis was further performed to characterize the functional distribution of identified DEGs. In leaves, a total of 5,693 DEGs were annotated to 315 KEGG pathways, among which 9 pathways were significantly enriched and upregulated at *P* < 0.05. The markedly enriched top 6 pathways primarily encompassed galactose metabolism (10/63), phenylalanine metabolism (7/35), starch and sucrose metabolism (17/144), biosynthesis of secondary metabolites (97/1,341), plant–pathogen interaction (29/302), and fatty acid biosynthesis (7/44). The values in parentheses indicate the ratio of differential genes to all background genes in each corresponding pathway (Test/Ref) (Figure 4C and Supplementary Table 4-4).

In root tissues, a total of 5,693 DEGs were annotated to 143 KEGG pathways, among which 26 pathways were significantly upregulated (*P* < 0.05). The top 10 significantly enriched pathways comprised biosynthesis of secondary metabolites (74/1,341), metabolic pathways (100/2,430), α-linolenic acid metabolism (13/57), phenylacetic acid biosynthesis (18/236), linoleic acid metabolism (4/12), plant MAPK signaling pathway (11/143), β-proline metabolism (6/45), cyanogenic acid metabolism (7/61), phenylalanine metabolism (5/35), and glycerol metabolism (7/71) (Figure 4D and Supplementary Table 4-5).

### 3.7 Verification of differentially expressed genes by qRT-PCR

Two significantly upregulated pathways, namely plant–pathogen interaction and phenylalanine metabolism, were selected for qRT-PCR validation in leaves. Two upregulated genes from each pathway, including CNGC1, MAPKKK1, PaA4G1 and PaA4G2, were screened to determine their relative expression levels. Under HIF treatment, the log2 (fold change) values of these four genes were 7.26, 3.03, 3.94, and 1.46, respectively. Correspondingly, their average relative expression levels quantified by qRT-PCR were 4.52 ± 0.52, 3.81 ± 0.96, 6.23 ± 0.34, and 5.81 ± 0.96. In summary, the expression upregulation trends of candidate genes identified via transcriptome analysis were highly consistent with the qRT-PCR results (Table 3).

**TABLE 3:** Verification of differentially expressed genes in the transcriptome by QRT-PCR.

KEGG pathway	Gene ID	Gene	Symbol	KO	log2 (FC)value *	P-adj	2^–ΔΔCT^ ± SD by HIF treated samples	2^–ΔΔCT^ ± SD of control samples
Plant-pathogen interaction	LOC102606712	*cngic*1	*CNGC* (cyclic nucleotide channel, plant)	K05391	7.26 up	6.15e-08	4.52 ± 0.52 up	1.19 ± 0.50
Plant-pathogen interaction	LOC102609173	*mapkkk*1	*MEKK*1 (mitogen-actived protein kinase kinase kinase 1)	K13414	3.03 up	3.09e-18	3.81 ± 0.96 up	1.82 ± 0.68
Phenylpropanoid metabolism	LOC112495556	*paA4g*1	E3.5.1.4, *amiE*	K01426	3.94 up	1.59e-07	6.23 ± 0.34 up	1.66 ± 0.32
Phenylpropanoid metabolism	LOC102625005	*paA4g*2	E3.5.1.4, *amiE*	K01426	1.46 up	0.04	5.81 ± 0.96 up	1.80 ± 0.60

*P < 0.05 and |log2(foldchange)| > 1 were used as the criteria for screening DEG. E3.5.1.4, *paA4g*1 was *amiE* (Acetamide hydrolase), it is involved in the carbon and nitrogen metabolism balance and affects the formation of biological membranes.

### 3.8 TMT-based pathway enrichment analysis of differential proteins

**FIGURE 5:**
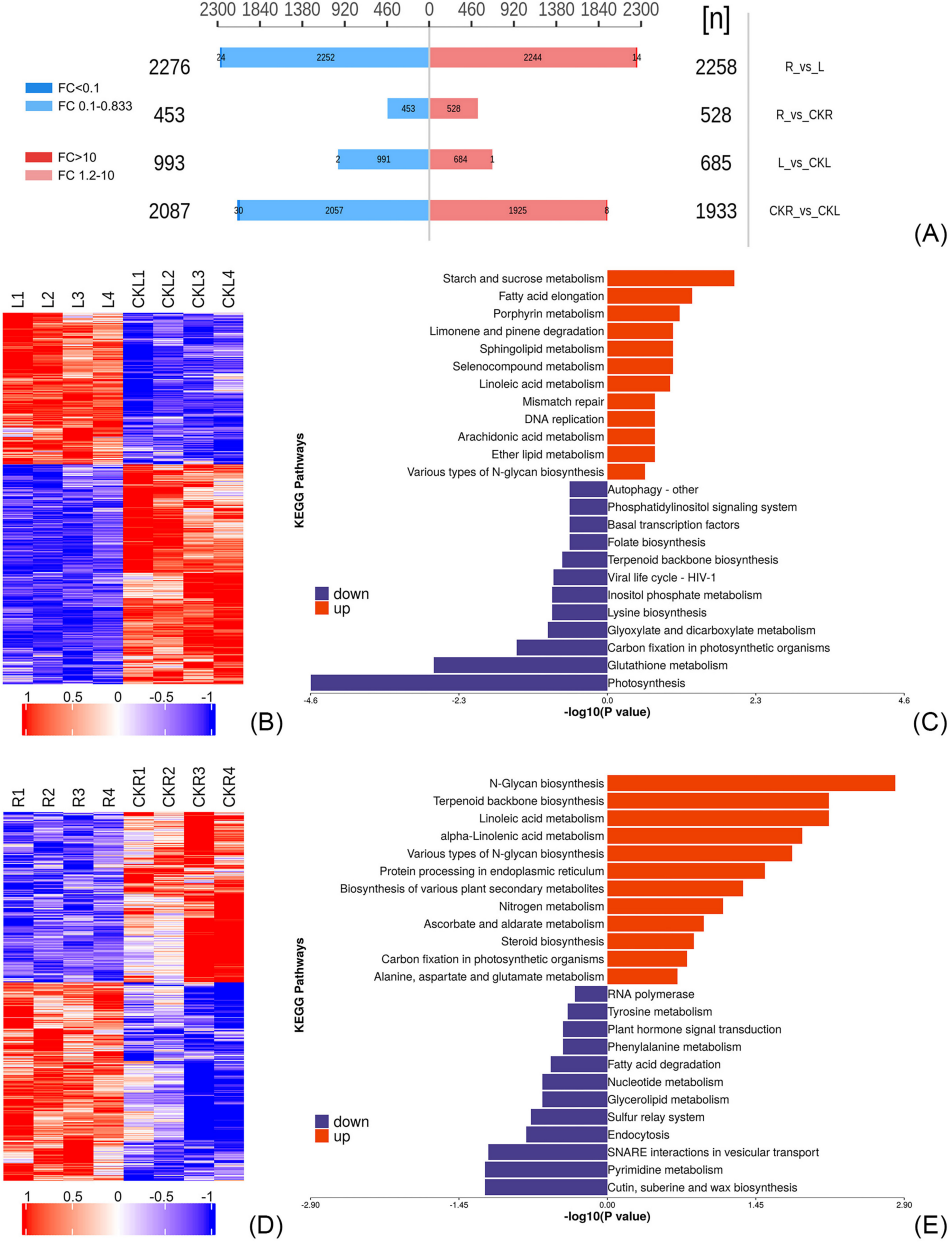
Proteomics analysis results. **(A)** Bar chart showing the numbers of differentially expressed proteins. **(B,D)** Cluster analysis of differentially expressed proteins in leaves and roots, respectively. **(C,E)** Butterfly plots showing pathway enrichment of top 12 up- and down-regulated proteins in leaves and roots, respectively.

To investigate the dynamic alterations of differentially expressed proteins (DEPs) in response to HIF treatment, TMT-based quantitative proteomic analysis was conducted in this study. Consequently, a total of 7,113 qualified proteins were accurately quantified. When compared with the control group, 685 proteins were significantly up-regulated and 993 proteins were significantly down-regulated in leaf tissues (*p* < 0.05). In root tissues, 528 and 453 proteins were significantly up-regulated and down-regulated (Figure 5A), respectively. Furthermore, hierarchical clustering analysis was separately implemented for DEPs in leaves and roots (Figures 5B,D and Supplementary Tables 5-1, 5-2).

In leaves, the enriched pathways corresponding to the upregulated differentially expressed proteins (DEPs) were as follows: starch and sucrose metabolism, fatty acid elongation, porphyrin metabolism (*P* < 0.05) (Figure 5C and Supplementary Table 5-3). In roots, the enriched pathways associated with upregulated DEPs included N-glycan biosynthesis, terpenoid backbone biosynthesis, linoleic acid metabolism, alpha-linolenic acid metabolism, protein processing in endoplasmic reticulum, biosynthesis of various plant secondary metabolites, nitrogen metabolism, the seven pathways showed significant differences (*P* < 0.05) (Figure 5E and Supplementary Tables 5-4).

### 3.9 Non-targeted metabolic changes following HIF treatment

**FIGURE 6:**
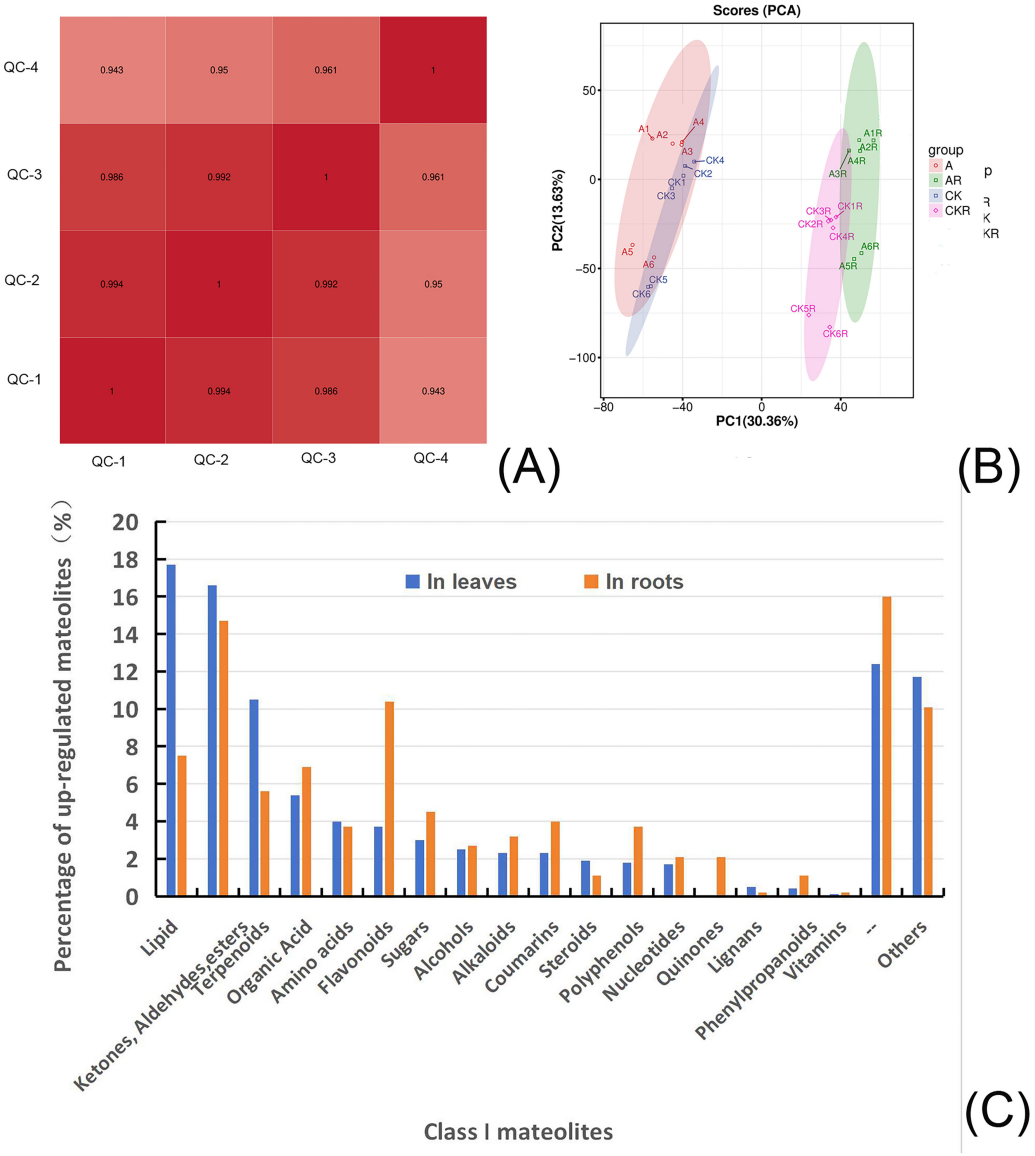
Quality control and the proportion of up-regulated metabolites in citrus. **(A)** Quality Control (QC) correlation heatmap. **(B)** Principal component analysis (PCA). **(C)** The proportion of up-regulated metabolites in leaves and roots.

PCA score plot indicated that the total variance explained was 13.63% for PC1, 30.36% for PC2, respectively. This conclusion was consistent with the QC correlation heatmap (all *R*^2^ > 0.942), demonstrating high reliability of the quality control (Figures 6A,B). After ten rounds of HIF treatment, a total of 5,101 metabolites were identified in plant leaves and roots. In leaves, 375 metabolites were significantly upregulated and 551 were downregulated, whereas 724 upregulated and 718 downregulated metabolites were detected in roots (Supplementary Tables 6-1–6-3).

The classification of major Class I upregulated metabolites in leaves and roots is listed below, respectively: Lipids (128, 17.7% vs. 28, 7.5%); ketones and aldehydes (120, 16.6% vs. 55, 14.7%); terpenoids (76, 10.5% vs. 21, 5.6%); amino acids (39, 5.4% vs. 26, 6.9%); flavonoids (27, 3.7% vs. 39, 10.4%); sugars (22, 3.0% vs. 17, 4.5%); alcohols (18, 2.5% vs. 10, 2.7%); alkaloids (17, 2.3% vs. 12, 3.2%); coumarins (17, 2.3% vs. 15, 4.0%); steroids (14, 1.9% vs. 4, 1.1%); polyphenols (13, 1.8% vs. 14, 3.7%); nucleotides (13, 1.7% vs. 8, 2.1%); quinones (7, 0.9% vs. 8, 2.1%); lignans (4, 0.5% vs. 1, 0.2%); phenylpropanoids (3, 0.4% vs. 4, 1.1%); vitamins (1, 0.1% vs. 1, 0.2%); other compounds (85, 11.7% vs. 38, 10.1%); and unknown metabolites (90, 12.4% vs. 38, 10.1%). The screening thresholds were set as fold change ≥ 2 and *P* < 0.05 for significantly differential metabolites (Figure 6C and Supplementary Tables 6-2, 6-3). The metabolic pathways involving the metabolites, as shown in Supplementary Tables 6-4, 6-5.

### 3.10 Metagenomics reveals bacterial diversity, correlation networks and AMP profiles of HIF

Raw reads were filtered using fastp software to obtain clean data. Species annotation at multiple taxonomic levels revealed that the microbial community in the HIF comprised 5 domains, 190 phyla, 168 classes, 331 orders, 736 families, 3,348 genera, and 20,626 species (Supplementary Tables 7-1, 7-2).

**FIGURE 7:**
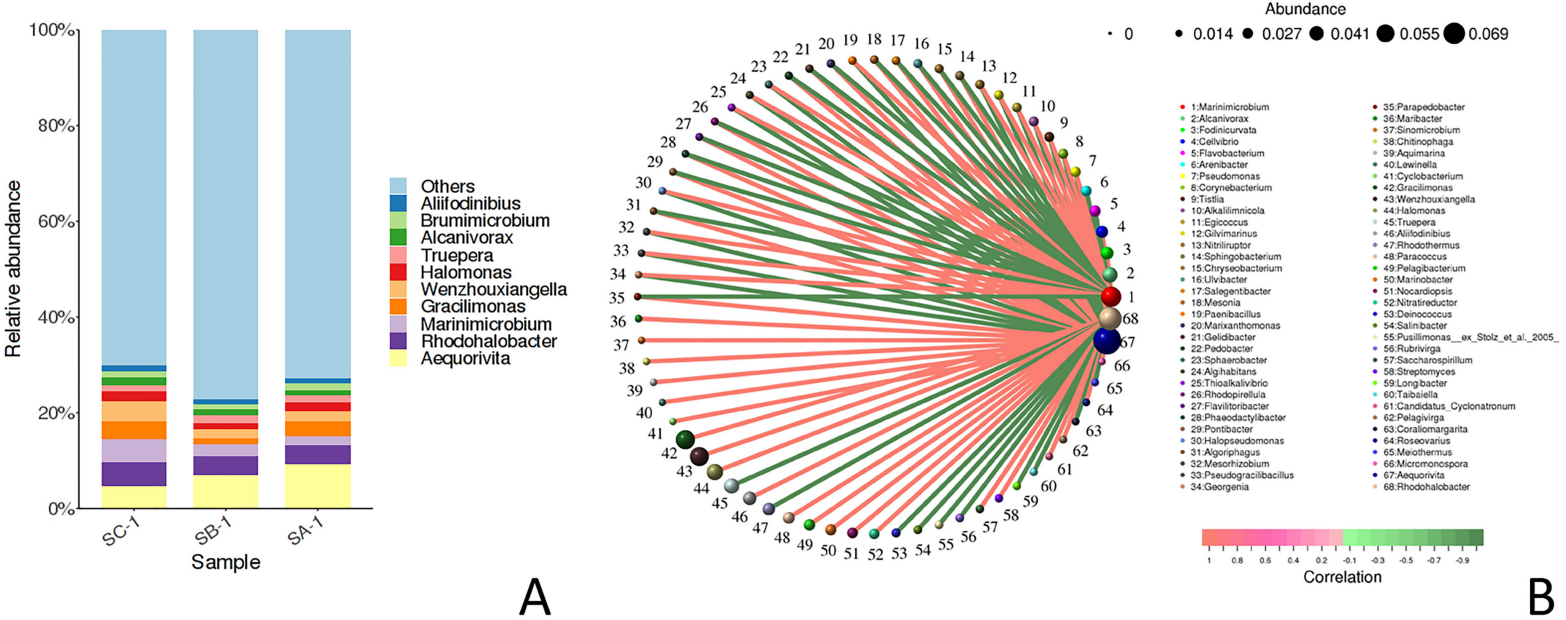
Metagenomic analysis results. **(A)** The top 10 genera in terms of abundance in HIF and correlation network diagram. **(B)** The circles represent species, and the size of the circles indicates the abundance; the thickness of the lines represents the strength of the correlation, and the color of the lines: red indicates a positive correlation, and green indicates a negative correlation.

At the genus level, the stacked bar chart intuitively reflected the microbial composition of each sample. The predominant genera included *Aequorivita* (6.85%), *Rhodohalobacter* (4.35%), *Marinimicrobium* (3.09%), *Gracilimonas* (2.69%), *Wenzhouxiangella* (2.63%), *Halomonas* (1.76%), *Truepera* (1.43%), *Alcanivorax* (1.38%), *Brumimicrobium* (1.31%), and *Aliifodinibius* (1.01%). Additionally, unclassified microorganisms, unassigned taxa, and other rare genera accounted for 24.42, 15.13, and 33.94% of the total community (Figure 7A and Supplementary Table 7-3), respectively.

The 68 most abundant genera were selected, and Spearman’s correlation analysis was performed based on the abundance and variation of each genus across all HIF samples. After statistical testing, a correlation network was constructed (Figure 7B and Supplementary Table 7-4). Among these genera, *Marinimicrobium*, *Aequorivita* and *Rhodohalobacter* served as three core network nodes, exhibiting close correlative relationships with 35, 41, and 25 other genera, respectively.

Protein sequences derived from the HIF Metagenome were aligned against NCBI reference sequences via BioEdit software to annotate antimicrobial peptides (AMPs). In total, 467 AMPs were identified, mainly consisting of 285 cecropins, 73 attacins, and 109 defensins (Table 4 and Supplementary Table 8).

**TABLE 4:** Types of AMPs in HIF.

Antimicrobial_peptide	Number
Attacin-a-like	25
Attacin-b-like	48
Defensin-like	67
Defensin-a-like	13
Defensin-a-likeisoformx	26
Defensin-b-like	3
Cecropin-likepeptide	273
Cecropin	7
Cecropin-b	5
Total	467

### 3.11 Non-targeted metabolites in HIF

**FIGURE 8:**
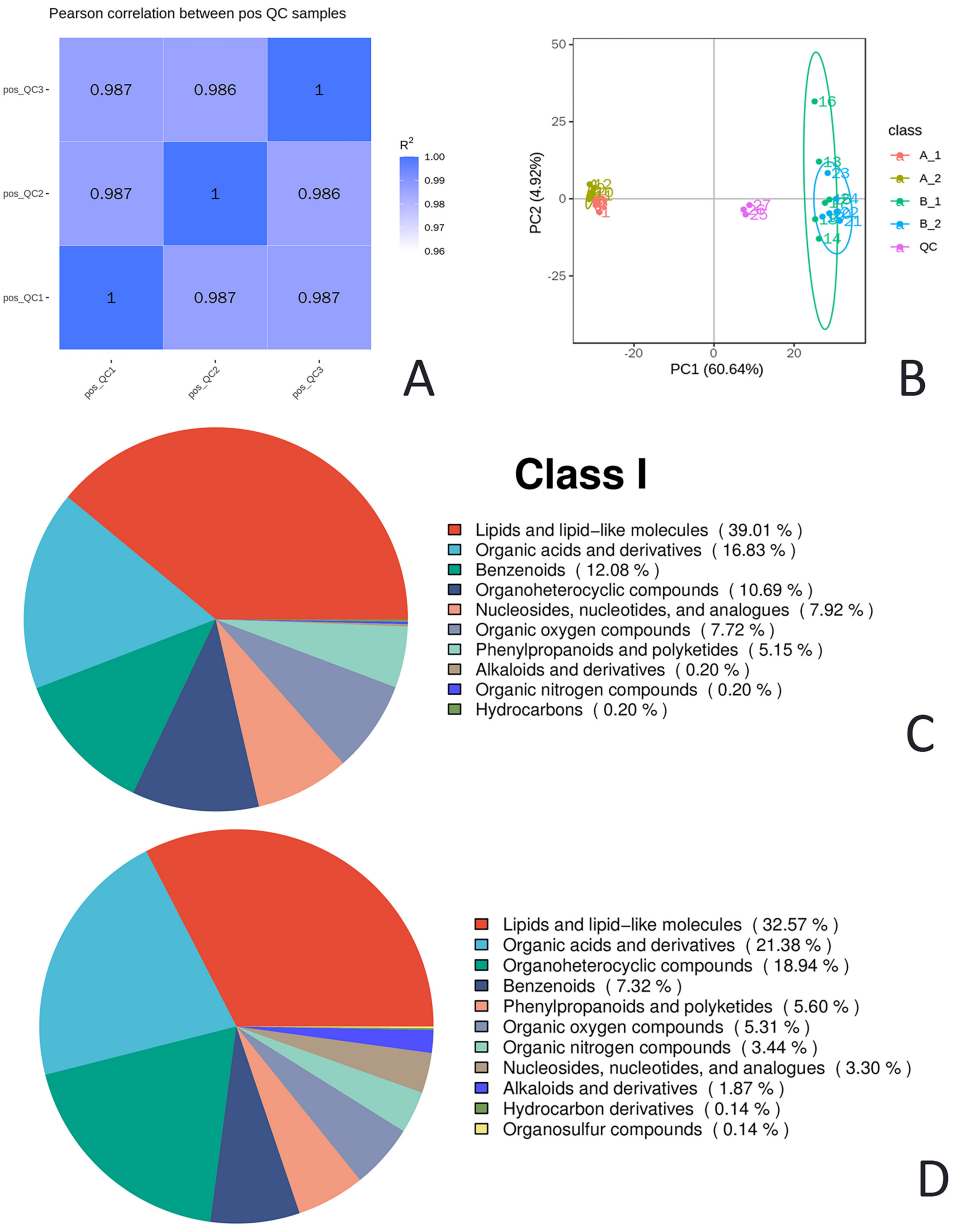
Quality control and pie chart of metabolite classes in HIF. **(A)** Correlation heatmap. **(B)** Principal component analysis (PCA). **(C,D)** The proportion of the metabolites (NEG & POS).

Correlation heatmap (all *R*^2^ > 0.98) demonstrating high reliability of the quality control dataset. The total variance explained by each PCA was 4.92% for PC1, 60.64% for PC2, respectively, indicating good explanatory power of the model (Figures 8A,B). Non-targeted metabolomics was applied to classify the detected metabolites and quantify their distribution across different categories (Figures 8C,D). A total of 1,301 metabolites in positive ion mode and 742 metabolites in negative ion mode were identified from the duplicated samples. The main metabolite categories and their corresponding proportions are listed as follows: lipids and lipid-like molecules (39.01, 32.57%), organic acids and derivatives (16.83, 21.38%), benzenoid compounds (12.06, 7.32%), organic heterocyclic compounds (10.69, 18.94%), nucleosides, nucleotides and analogs (7.92, 3.30%), organic oxygen compounds (7.72, 5.31%), as well as phenylpropanoid and polyketide compounds (5.15, 5.60%) (Supplementary Table 9). The two percentages in each parenthesis correspond to the data obtained from positive and negative ion modes, respectively. These metabolites are closely associated with plant disease resistance as well as fundamental physiological and biochemical processes.

## 4 Discussion

To develop an efficient and durable strategy for controlling citrus HLB, we explored novel microbial fertilizer, HIF to *C*Las-infected plants in both 20 and 25 treatments, which resulted in significant reduction in *C*Las titer. HIF promoted growth by stimulating robust root development and reversing leaf chlorosis, the contents of auxin, cytokinin and salicylic acid (SA), an immune signal molecule, was significantly upregulated after HIF applications; Top 10 high abundance genera were found in flora analysis in citrus tissues, most of them refer to plant growth promoting effect. Integrated analyses of transcriptome–metabolome, transcriptome–proteome, and proteome–metabolome jointly identified 15, 4, and 1 co-upregulated KEGG pathways, respectively. Meanwhile, α-linolenic acid metabolism was consistently and significantly upregulated as discovered by all three omics. Furthermore, 467 AMPs, cyclopeptide, abundant metabolites, and the beneficial bacterial community in HIF, which provided the clues for *candidate* verification of Huanglongbing resistance.

### 4.1 HIF induced content of SA increasing in citrus

Pathogen-associated molecular patterns (PAMPs) can initiate pattern-triggered immunity (PTI), activate the plant cellular immune response, enabling effective resistance to pathogens (Yu et al., 2022). Beneficial microorganisms can also elicit plant-ISR. Upon ISR activation, plants use long-distance signals to confer protection to distal tissues, promote growth, and resist pathogen invasion (Alstrom, 1991; Gang et al., 1991; Vanpeer et al., 1991). In this study, HIF treatment significantly increased SA by 3.03-fold in leaves, the finding suggest that HIF possibly enhances systemic resistance (SAR) in citrus, improving resilience against HLB. Previous studies have shown that Citrus activates *C*Las defense through xylem cysteine peptidase 2-mediated CsPR1 cleavage, and amplify SA signaling, exogenous SA decreases *C*Las loads in infected citrus (Hu et al., 2026). Here, HIF treatment raised leaf SA concentrations, yet the link between elevated SA and CsPR1 production requires further research.

### 4.2 Up-regulation beneficial microorganisms may promote the growth in cirus

HIF treatment significantly increased the abundance of *Allorhizobium_Neorhizobium_Pararhizobium_Rhizobium* (ANPR) and *Serratia* in both leaves and roots. Meanwhile, *Acinetobacter, Sphingomonas*, and *Actinomycetospora* were enriched in leaves, while *Streptomyces* were markedly accumulated in roots relative to the control.

These genera have well-documented ecological functions in previous research. *Serratia marcescens* shows antagonistic activity against *Staphylococcus aureus* (Chen et al., 2025). *Allorhizobium_Neorhizobium_Pararhizobium_Rhizobium* (ANPR) serves as a core microbiome member linked to the growth and medicinal quality of lily bulbs and ginseng, and alleviates continuous cropping obstacles (Kozma Kim et al., 2024; Wang and Hou, 2025). *Sphingomonas* possesses strong metabolic versatility, with capabilities in exopolysaccharide secretion, biofilm formation and organic pollutant degradation (Liu et al., 2023). As typical PGPR, *Streptomyces* exhibits prominent biocontrol potential; its strain AC-3 strongly inhibits oomycetes causing *tomato late blight* (Suárez-Fernández et al., 2026).

### 4.3 Metabolites could become the main force of the anti-*C*las

A wide range of specialized metabolites including coumarins, flavonoids, phenylpyrroles, sesquiterpenes, polyketides, Liliaceae, and alkaloids derivatives exhibit antioxidant, antibacterial, antifungal, and antiviral defense functions via distinct molecular mechanisms against various pathogens (Yang et al., 2026; Ruan et al., 2026; Leonarduzzi et al., 2010; Wang et al., 2025; Abdelfattah, 2009; Ji et al., 2016). Upregulated metabolites form a multi-level plant defense network through direct antimicrobial effects, immune signal regulation, reactive oxygen species scavenging, and pathogen metabolism interference. Recent studies show MYC2-dependent JA signaling enhances citrus HLB resistance via accumulating antimicrobial isoprenoids, flavonoids and phenylpropanoids to defend against CLas (McCartney et al., 2024). In this study, significant upregulation was also observed for 10 isoprenoid, 39 flavonoid, 6 phenylpropanoid and 26 organic acid in citrus leaves, 39 organic acid in roots following HIF treatment (Supplementary Table 6).

Transcriptomic analysis of susceptible and resistant citrus shows resistant varieties trigger no typical immune responses to *C*Las, with few differentially expressed genes (DEGs). Instead, they remodel primary metabolism and core cellular functions to form an unfavorable microenvironment for *C*Las colonization and proliferation (Alves et al., 2025). In our experiment, HIF treatment applied in the present study induces widespread upregulation of numerous metabolites, which potentially contributes substantially to the inhibition of *C*Las growth and propagation. Here, both citrus leaves and roots demonstrates that HIF initiates a systematic and multi-layered chemical changes. In aboveground tissues, elevated levels of flavonoids, polyphenols, terpenoids and lipids reinforce foliar immunity as well as physical and chemical barriers. In belowground roots, more intensive metabolic remodeling occurs, particularly in lipids, terpenoids, carbohydrates and coumarins.

### 4.4 Key genes expression trends are consistent with the transcriptome

CNGC1-mediated Ca^2+^ signaling and MAPKKK1-involved MAPK cascade can activate the early immune signaling pathway and defense signal transduction. Meanwhile, the significant up-regulation of PaA4G1 and PaA4G2 promotes the biosynthesis of phenylpropanoid-derived metabolites such as lignin, phenolics and phytoalexins (Kong et al., 2012; Bi et al., 2018). The expression trends of key genes *CNGC1*, *MAPKKK1, PaA4G1*, and *PaA4G2* were verified by qPCR and were found to be highly consistent with the transcriptome here, whether HIF can genuinely activate citrus innate immunity and defense signal transduction remains to be verified.

### 4.5 Co-upregulated transcriptomic and proteomic pathways post HIF treatment uncover new clues for HLB resistance

Reviewing the common pathways shared by the transcriptome and proteome, it is not difficult to discover that the simultaneous upregulation of starch and sucrose metabolism in the leaves, providing sufficient energy and carbon skeletons for defense responses; while the enhanced amino sugar and nucleotide sugar metabolism directly promotes the synthesis of cell wall polysaccharides and suberin, strengthening the physical barrier and activating PTI immunity, constituting the core disease resistance mechanism of the leaves. The linoleic acid metabolism pathway specifically upregulated in the root, as an important upstream pathway for jasmonic acid and oxylipin synthesis, participates in root-based defense and systemic resistance signal transmission, achieving coordinated regulation of disease responses in the underground and aboveground parts (Table 5).

**TABLE 5:** Combined analysis of transcriptome, proteome and metabolome.

Leaves	Description of KEGG	Transcriptome		Proteomics		Metabolomics		Metabolite_ID
Ko-ID		Test/Ref*	*P*-value	Test/Ref #	*P*-value	Diff_metabolites_ in_pathway	All_metabolites_ in_pathway	
ko00500	Starch and sucrose metabolism	17/144	0.0023	5/15	0.0107	−	−	−
ko00520	Amino sugar and nucleotide sugar metabolism	14/155	0.046992	4/14	0.0389	−	−	−
ko00052	Galactose metabolism	10/63	0.0021			1	4	neg7248
ko00360	Phenylalanine metabolism	10/63	0.0021			1	2	neg2827
ko00061	Fatty acid biosynthesis	7/44	0.0097			1	1	neg3117
ko00073	Cutin, suberine and wax biosynthesis	4/27	0.0590			2	3	pos5859, pos8974
ko00350	Tyrosine metabolism	7/64	0.0618			2	6	pos4799, pos1734
ko00950	Isoquinoline alkaloid biosynthesis	5/35	0.0511			2	5	pos6241, neg6379
ko02010	ABC transporters	4/25	0.0463			2	17	pos4107; neg3760
Roots								
ko00592	alpha-Linolenic acid metabolism	13/57	8.70E-10	4/13	0.0125	3	3	pos4424, neg8346, neg6553
ko00940	Phenylpropanoid biosynthesis	18/236	2.1192e-05			1	5	neg6209
ko00410	beta-Alanine metabolism	6/45	0.0008			1	2	neg3760
ko00460	Cyanoamino acid metabolism	7/61	0.0007			2	5	neg3760, pos975
ko00260	Glycine, serine and threonine metabolism	6/67	0.0064			2	5	pos7565, neg3760
ko00052	Galactose metabolism	5/63	0.0204			1	4	neg1004
ko00630	Glyoxylate and dicarboxylate metabolism	5/65	0.0230			3	9	Pos5702, neg3760, neg4075
ko00910	Nitrogen metabolism	3/31	0.0417			1	1	neg4075
ko00770	Pantothenate and CoA biosynthesis	3/37	0.0648			1	3	neg3760
ko00591	Linoleic acid metabolism	4/12	0.0001	3/6	0.0068	−		
ko00900	Terpenoid backbone biosynthesis	5/62	0.0191	3/6	0.0068			
ko00999	Biosynthesis of other secondary metabolites			2/5	0.0473	5	42	neg_7370; pos_2997; pos_7409; neg_3760; neg_5728

*The values indicate the ratio of differential genes to all background genes in each corresponding pathway (Test/Ref). ^#^The values indicate the ratio of differential proteins to all background proteins in each corresponding pathway.

HLB infection induces upregulation of flavonoid biosynthesis and phenylpropanoid pathway genes in tolerant plants, enhancing antioxidant ability and cell wall strength (Hendrich et al., 2025). This observation aligns with the upregulated pathways identified via transcriptomic analysis under HIF treatment in the present study.

The co-upregulated KEGG pathways identified via multi-omics analyses can only demonstrate correlation, rather than direct causality. These pathways serve merely as correlative evidence linking three variables: Huanglongbing (HLB) stress, exogenous HIF treatment, and disease-resistant phenotypes. Their synchronous activation alongside disease-resistant traits merely indicates concurrence; collective upregulation of these pathways does not substantiate that pathway activation directly mediates plant disease resistance. Further stratified validation assays are therefore required in follow-up research, for example, by gene knockout or pathway inhibition.

### 4.6 Transcriptional regulation–metabolic phenotype underlying HLB resistance

Integrated transcriptome and metabolome data reveal possible defensive pathways in citrus leaves and roots. Leaf pathways build physical barriers, produce antimicrobial metabolites and supply energy via lignin, flavonoid, cuticular lipid and ABC transporter systems. Root pathways reinforce cell walls, balance carbon-nitrogen and amino acid metabolism, and maintain long-lasting immunity. Coordinated multi-omics shifts verify genuine defense activation instead of single-omics false signals (Table 5). Alpha-linolenic acid metabolism, a universally upregulated core immune hub, generates JA precursor oxylipins that suppress pathogens directly and induce systemic acquired resistance through SA-JA crosstalk (Seth et al., 2024; Chang and Lv, 2024). The above phenomena provide a train of thought for further verification.

### 4.7 Beneficial microbes in HIF could improve rhizosphere health

Previous studies have confirmed the presence of *Lactobacillus, Actinobacteria, and Bacillus* in *black soldier fly* manure (Zhang et al., 2023; Tegtmeier et al., 2021). In this study, some beneficial microbes in HIF were uncovered. These dominant genera are generally halo-stress adaptable, functioning in organic decomposition, nutrient mineralization and rhizosphere microenvironment regulation (Lukhele et al., 2022; Ara et al., 2020). As a core beneficial microbe, *Halomonas* facilitates root growth, and triggers plant systemic resistance (Dindhoria et al., 2025). *Alcanivorax* drives lipid degradation and carbon metabolism (Tiwari et al., 2025). *Truepera* maintains redox balance via oxidative stress resistance, and other core taxa enhance organic fertilizer mineralization, nutrient supply and community stability (Albuquerque et al., 2005). Collectively, these key microbes modulate host immunity and metabolism, thereby may improve citrus growth and adaptability.

### 4.8 Multiple genera in HIF having potential growth promoting functions

The network analysis of HIF in Metagenome has concluded that 68 high-abundance genera, numerous of which have plant growth-promoting (PGP) traits, including *Flavobacterium, Pseudomonas, Sphingobacterium, Chryseobacterium, Paenibacillus, Rhodopirellula, Pontibacter, Mesorhizobium, Chitinophaga, and Halobacillus.*

Previous studies have verified: *Flavobacterium pokkalii* strain L1I52T promotes Pokkali rice growth (Menon et al., 2020); quinoa endophytic *Pseudomonas* enhances salt tolerance and root architecture of *Arabidopsis thaliana* (Ruiz et al., 2026); *Sphingobacterium kyonggiense* T8 remediates antibiotic-nitrogen co-polluted environments (Leng et al., 2026); *Chryseobacterium oleae* induces olive cutting rooting (Montero-Calasanz et al., 2014); *Paenibacillus elgii* YSY-1.2 shows chitinolytic, PGP and biocontrol activities (Tran et al., 2024); *Burkholderiaceae* and *Rhodopirellula* facilitate phosphate solubilization and mineralization (Ortega et al., 2024); *Pontibacter niistensis* NII-0905 has multiple PGP characteristics (Dastager et al., 2011); wheat rhizosphere *Pseudomonas* strains are potential plant growth-promoting rhizobacteria (PGPR) (Todorović et al., 2023); *Mesorhizobium sp*. combined with PGPR improves chickpea nodulation, growth and yield (Verma et al., 2013); phosphate-solubilizing bacteria antagonize *Fusarium oxysporum* and promote plant growth (Zhou et al., 2025); *Halobacillus marinus* SL2 enhance wheat salt tolerance and growth (Dindhoria et al., 2025).

### 4.9 Both cyclic- lipopeptides and AMPs in HIF form the possible dual defensive barrier

Beneficial microorganisms secrete various natural antimicrobial substances, among which Cyclic-lipopeptides are an important class (Zeriouh et al., 2014; Hamley, 2015). The lipophilic character of surfactins enables them to incorporate into cell membranes, disrupting pathogen structure and function and causing cell death. Additionally, the hydroxy fatty acid structure of surfactins can activate plant immunity, contributing to durable protection (Qiao et al., 2024; Baindara et al., 2013). According to laboratory HPLC analyses methods (Ding et al., 2024), HIF contained 175.85 mg/kg of surfactin, as well as detectable 28.764 (mg/kg) iturin, with a mean total lipopeptide concentration of 204.61 mg/kg (Table 6 and Supplementary Figure 5).

**TABLE 6:** Concentration of lipopeptides in HIF with HPLC.

Lipopeptides	Number of samples	Retention time	Peak area	Concentration (mg/L)	Average concentration (mg/kg)
Iturin*	SS1	3.208	1,645,000	49.971	28.764 ± 14.997
	SS2	3.149	586,718	18.404	
	SS3	3.146	570,404	17.917	
Surfactin^#^	S1 (peak 1)	13.377	20,919	41.128	44.887 ± 5.435
	S2 (peak 1)	13.537	180,829	52.573	
	S3 (peak 1)	13.404	18,562	40.959	
	S1(peak 2)	13.893	6,105	21.986	21.981 ± 0.005
	S2 (peak 2)	13.934	5,672	21.976	
	S1 (peak 3)	14.669	2,756	46.721	46.649 ± 0.072
	S2 (peak 3)	14.685	1,810	46.577	
	S1 (peak 4)	15.481	19,480	60.661	62.328 ± 1.667
	S2 (peak 4)	15.499	33,024	63.994	
Total					204.61

*One characteristic peak. ^#^Four characteristic peaks.

Cecropins, attacins and defensins are key innate immune antimicrobial peptides/proteins widespread in insects and other organisms (Zhao and Dai, 1999; Zhan et al., 2020; Manniello et al., 2021). Linear α-helical cecropins kill Gram-negative bacteria via membrane disruption and pore formation (Gimranov et al., 2025). Attacins target Gram-negative strains by binding lipopolysaccharides, damaging outer membranes and inhibiting outer membrane protein synthesis. Disulfide-crosslinked defensins show broad activity, these peptides have been utilized to manage rice bacterial blight and potato bacterial wilt (Jian et al., 1997; Jia et al., 1998). Existing literature on the application of peptides for Huanglongbing (HLB) disease resistance predominantly reports findings derived from fundamental laboratory investigations. In-depth research under actual field conditions and upon infection by diverse pathogenic strains remains insufficient. In this study, Metagenomic sequence alignment identified 467 antimicrobial peptides in HIF. These three classes of molecules are possibly complementary in structure, antimicrobial spectrum and mechanism of action. Our experiment places specific emphasis on preserving and harnessing diverse microbial communities. Further investigations of HIF in orchards with diverse citrus species, HLB strains are required. Notably, chicken manure must be obtained from certified commercial farms and guaranteed free of heavy metal contamination.

**FIGURE 9:**
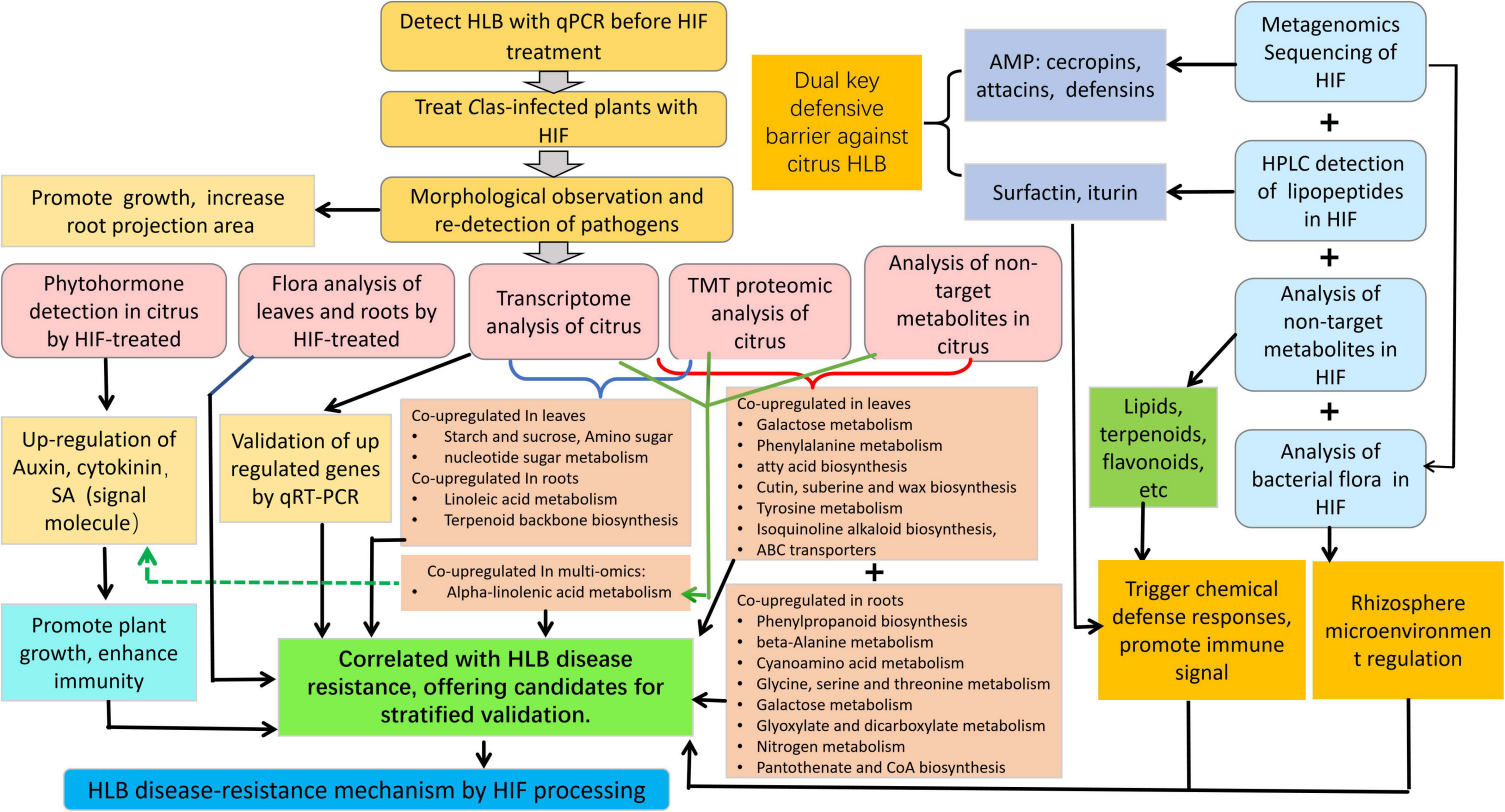
Candidate clues for HLB disease resistance factors and stratified validation.

In summary, this study presents an innovative strategy utilizing HIF to control Huanglongbing diseases to a certain degree and facilitate citrus plants rejuvenation. This constitutes a breakthrough in the field and merits further in-depth exploration. The present findings also provide Candidate clues for HLB disease resistance factors and stratified validation (Figure 9).

## Data Availability

The datasets presented in this study can be found online repositories. The name of repository and accession number(s) can be found in the article/Supplementary material.
