# Supplementary material for: Control efficiency and Huanglongbing resistance-related clues mediated by novel Hermetia illucens frass formulation
Source: Front Microbiol. 2026 Aug 4;17:1896630. doi: 10.3389/fmicb.2026.1896630 (PMC13481663; doi:10.3389/fmicb.2026.1896630)
Supplement: Supplementary file 1 [file Data_Sheet_1.zip › S Fig1-5/Fig. 1.pdf]

S Fig.1

Cloning vector construction

Primer 1 Name： RNR F/RNR R

Gene number： CP001677.5

Candidatus Liberibacter asiaticus str. psy62, complete genome

Design site： β-subunit of ribonucleotide reductase

TA cloning primers：

RNR F: CATGCTCCATGAAGCTACCC

RNR R: GGAGCATTTAACCCCACGAA

The amplified fragment of *RNR*：

CATGCTCCATGAAGCTACCCTCCTCGAAATCGCCTATGCACATGAAACAATGCCAAAAGGGTTCGTGGGGTT AAATGCTCC

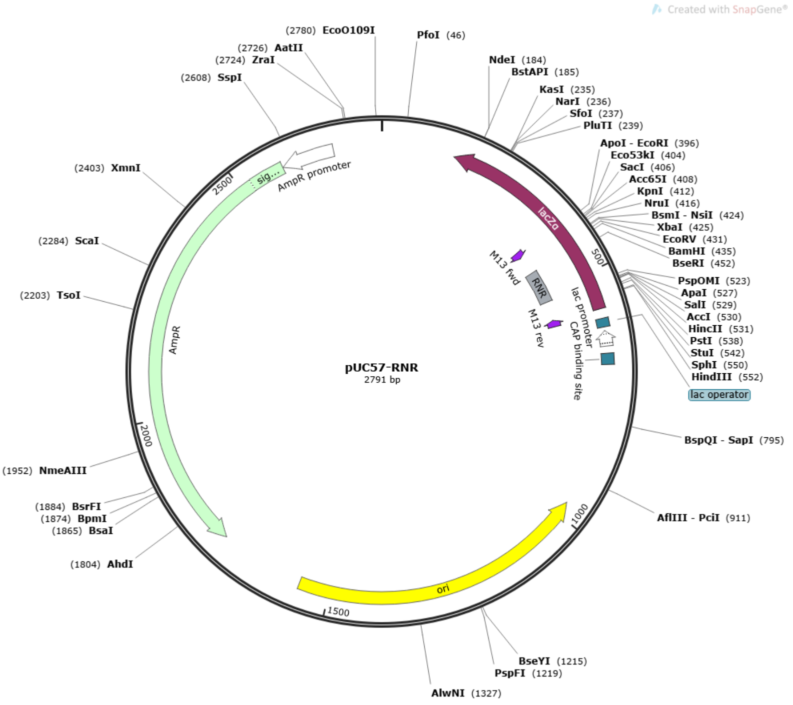

Primer 2 Name： COX F/COXR

Gene number： CX297817.1

Citrus clementina x Citrus tangerina cDNA clone C06005E09, mRNA sequence

TA cloning primers：

COX F: GTATGCCACGTCGCATTCCAGA

COX R: CCAAAACTGCTAAGGGCATTC

The amplified fragment of *COX*:

GTATGCCACGTCGCATTCCAGATTATCCAGATGCTTACGCTGGATGGAATGCCCTTAGCAGTTTTGG

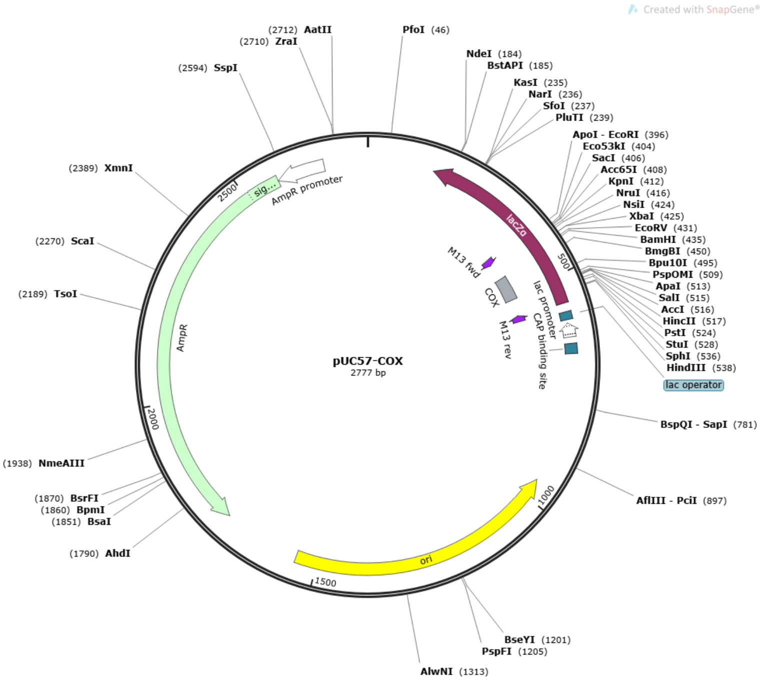

The map was created with SnapGene software.

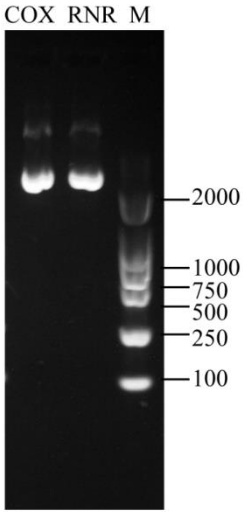

M: DL2000 Marker

All plasmids were extracted and tested by gel electrophoresis.
