# Supplementary material for: Control efficiency and Huanglongbing resistance-related clues mediated by novel Hermetia illucens frass formulation
Source: Front Microbiol. 2026 Aug 4;17:1896630. doi: 10.3389/fmicb.2026.1896630 (PMC13481663; doi:10.3389/fmicb.2026.1896630)
Supplement: Supplementary file 1 [file Data_Sheet_1.zip › S Fig1-5/Fig. 2.pdf]

**S Fig. 2**

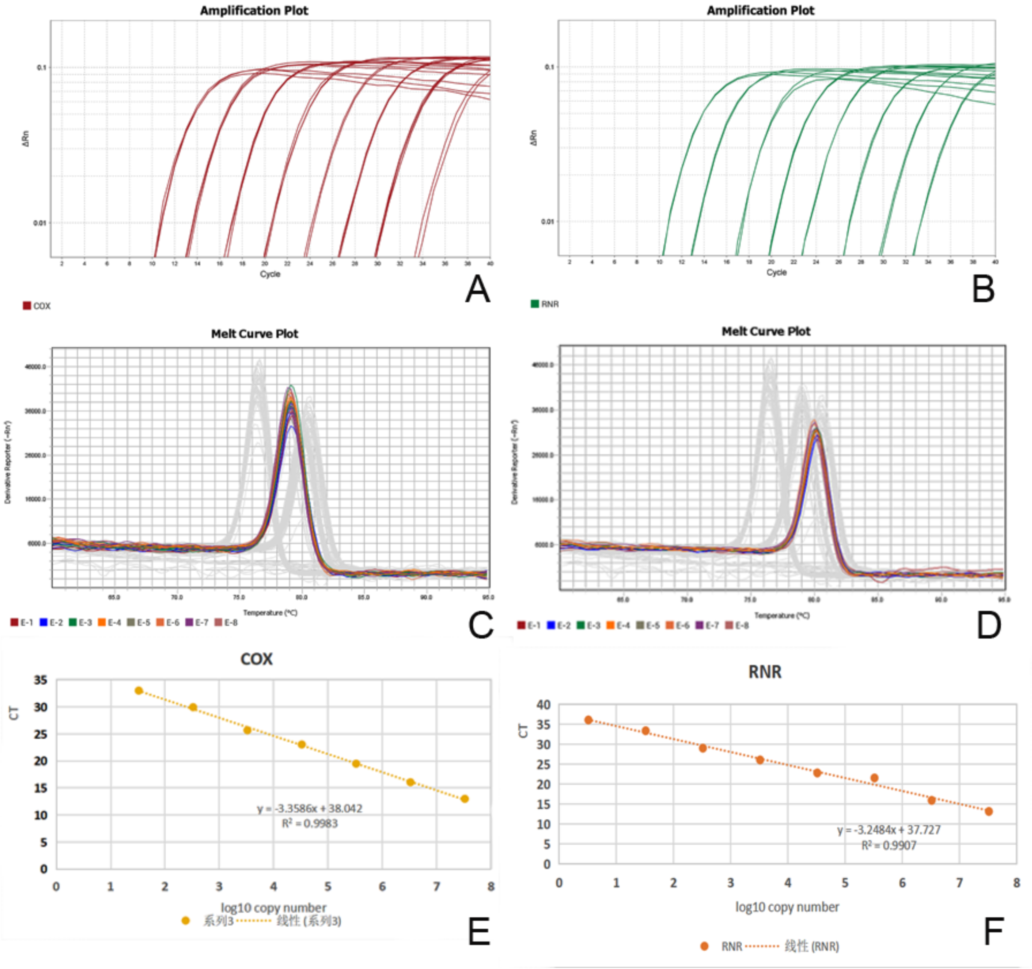


**S Fig. 2 Amplification curves, melting curves, and standard curve equations for the housekeeping gene (*COX*) and target gene (*RNR*).**

A, C & E: *COX* genes. B, D & F: *RNR* gene.
