# Supplementary material for: Control efficiency and Huanglongbing resistance-related clues mediated by novel Hermetia illucens frass formulation
Source: Front Microbiol. 2026 Aug 4;17:1896630. doi: 10.3389/fmicb.2026.1896630 (PMC13481663; doi:10.3389/fmicb.2026.1896630)
Supplement: Supplementary file 1 [file Data_Sheet_1.zip › S Fig1-5/Fig. 3.pdf]

S Fig.3 Statistical analysis results of changes in pathogen titers

Part 1

1. Descriptive Statistics

Each group contained six biological replicates.

Table 1 Changes in *C*las pathogen titers in citrus veins

Sample name	1	2	3	4	5	6
CK	8699.17	10534.06	712.51	7711.6	5645.38	5296.48
CK20	2428.62	3247.69	3663.6	7821.7	14389.54	10534.06
CK25	9013.01	8161.53	5075.94	4629.1	3004.08	1499.77

Note: Samples at three time points of CK, CK20 and CK25

Table 2 Mean value and sample variance

Sample name	Group Sample size	Mean value	Sample variance
CK	6	6433.20	11645013.23
CK20	6	7014.20	22775554.53
CK25	6	5230.57	8431836.37

2. One-way Analysis of Variance (One-way ANOVA)

A completely randomized single-factor ANOVA was performed to detect intergroup differences.
Total observations N=18, number of treatments k=3.
Degrees of freedom: df_{between}=k-1=2, df_{within}=N-k=15.

- Sum of squares between groups (SS_{between}) = 9930427.64
- Sum of squares within groups (SS_{within}) = 214262020.65
- Mean square between groups (MS_{between}) = SS_{between}/df_{between} = 4965213.82
- Mean square within groups (MS_{within}) = SS_{within}/df_{within} = 14284134.71
- F-value = MS_{between}/MS_{within} = 0.3476

The critical F-value at \alpha=0.05 for df_1=2, df_2=15 was 3.68. Since the calculated F=0.3476 < 3.68, there was no statistically significant overall difference among the three groups (P>0.05).

3. Multiple Comparisons

Post-hoc multiple comparisons (LSD or Tukey test) were not required, as the global ANOVA test showed non-significant intergroup variation. No significant mean difference was detected between any pair of groups.

Mean ranking: CK20 (7014.20) > CK (6433.20) > CK25 (5230.57). Although CK20 had the highest average value and CK25 the lowest, extremely large intra-group variation masked the minor intergroup discrepancy.

4. Conclusions

1. One-way ANOVA output: F=0.348, df_1=2, df_2=15, P>0.05.
2. No significant statistical differences in the target index were observed among CK, CK20 and CK25.
3. All pairwise comparisons between groups exhibited non-significant differences.
4. Substantial fluctuations existed within replicates of each group, indicating high experimental error. Increasing replicate numbers and optimizing experimental conditions are recommended to reduce random error for distinguishing potential treatment effects.

Letter Marking for Significance
CK20 a
CK a
CK25 a
Identical lowercase letter "a" shared by all groups indicates no significant difference between any two treatments.

Part 2

Step 1: Data Organization

The experiment included three groups with 6 replicates each:

Table 1 Changes in pathogen titers in citrus veins after HIF treatment

Sample name	1	2	3	4	5	6
Control (CK)	7234.99	7877.34	3536.03	4149.78	4899.19	3932.72
HIF20	1963.38	475.68	3294.06	2498.46	4312.33	2445.89
HIF25	485.91	775.77	1949.52	1310.79	4343.01	3201.97

Step 2: Calculation of Group Means

- Mean of CK ($\bar{x}_{CK}$): 5271.68
- Mean of HIF20 ($\bar{x}_{T1}$): 2498.30
- Mean of HIF25 ($\bar{x}_{T2}$): 2011.16

Step 3: One-Way Analysis of Variance (ANOVA)

- Sum of squares between groups ($SS_{between}$) = 18562359.84, degrees of freedom ($df_{between}$) = 2, mean square between groups ($MS_{between}$) = 9281179.92
- Sum of squares within groups (SS_{within}) = 36353016.65, degrees of freedom (df_{within}) = 15, mean square within groups (MS_{within}) = 2423534.44
- F-value = $MS_{between}/MS_{within}$ = 3.83

The critical F-value at $\alpha=0.05$ with $df_1=2$ and $df_2=15$ is 3.68. Since the calculated F-value (3.83) > 3.68, there was a significant difference among the three groups ($P<0.05$).

Step 4: Post-Hoc Multiple Comparisons (LSD Test)

- Least significant difference (LSD) at $\alpha=0.05$ was calculated as:

$$LSD_{0.05} = t_{0.05(15)} \times \sqrt{\frac{2MS_{within}}{n}} = 2.131 \times \sqrt{\frac{2 \times 2423534.44}{6}} \approx 1914.5$$

- Comparisons of mean differences:
- CK vs.HIFi20: 5271.68 - 2498.30 = 2773.38 > 1914.5 → significant difference ($P<0.05$)
- CK vs. HIFi25: 5271.68 - 2011.16 = 3260.52 > 1914.5 → significant difference ($P<0.05$)
HIFi20 vs. HIFi25: 2498.30 - 2011.16 = 487.14 < 1914.5 → no significant difference ($P>0.05$)

Conclusion

One-way ANOVA revealed a significant overall difference among the three groups ($F=3.83$, $P<0.05$). Post-hoc LSD tests showed that CK was significantly different from both HIF20 and HIF25, while no significant difference was detected between HIF20 and HIF25.
