# Supplementary material for: Control efficiency and Huanglongbing resistance-related clues mediated by novel Hermetia illucens frass formulation
Source: Front Microbiol. 2026 Aug 4;17:1896630. doi: 10.3389/fmicb.2026.1896630 (PMC13481663; doi:10.3389/fmicb.2026.1896630)
Supplement: Supplementary file 1 [file Data_Sheet_1.zip › S Fig1-5/Fig. 4.pdf]

S Fig. 4 XIC of hormone standard samples and sample to be tested

The XIC graph of the standard samples provided by Novogene Co., Ltd for this experiment

S Fig. 4-1 XIC of hormone standard samples

STD1

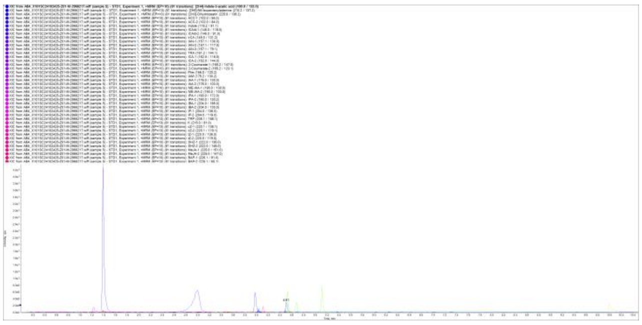

STD2

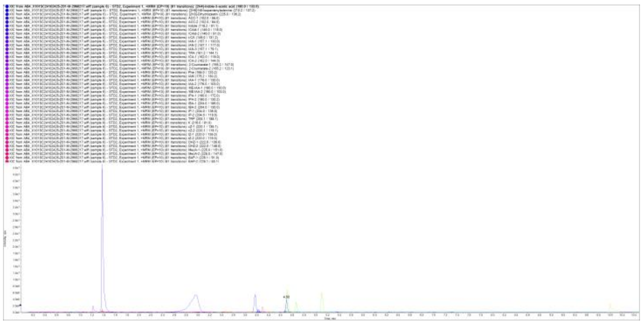

STD 3

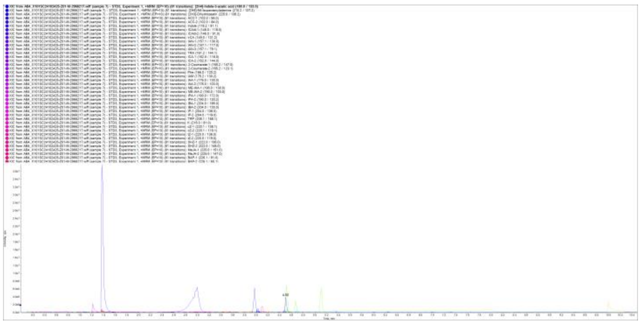

STD 4

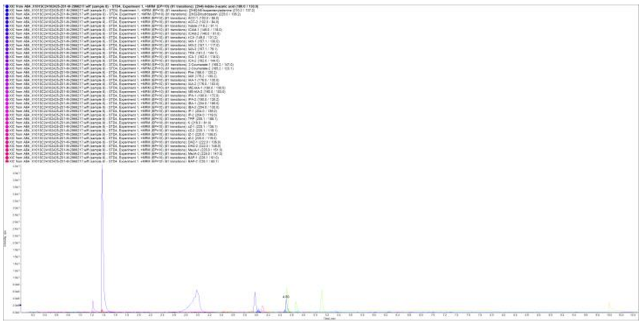

STD5

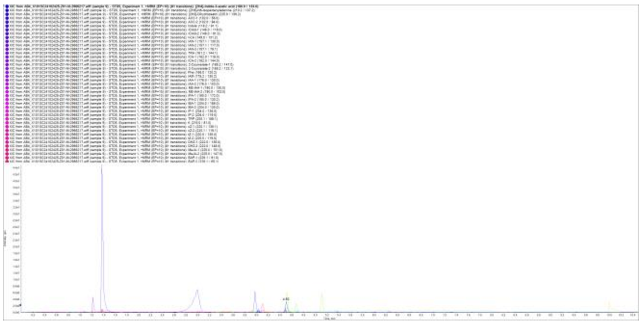

STD6

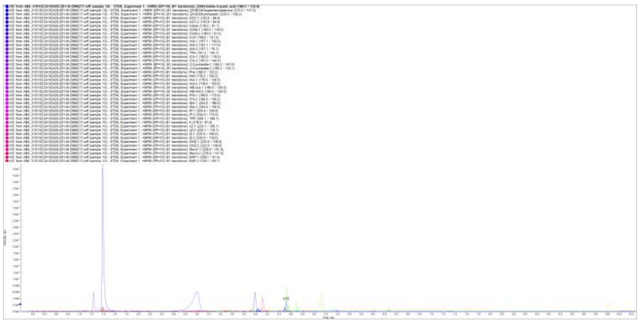

STD7

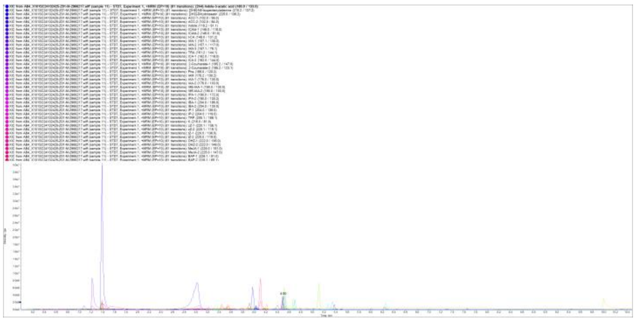

STD8

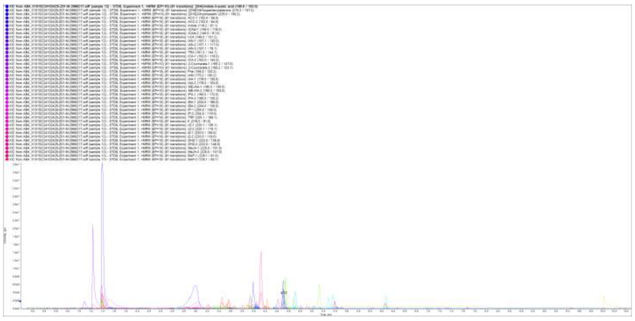

STD9

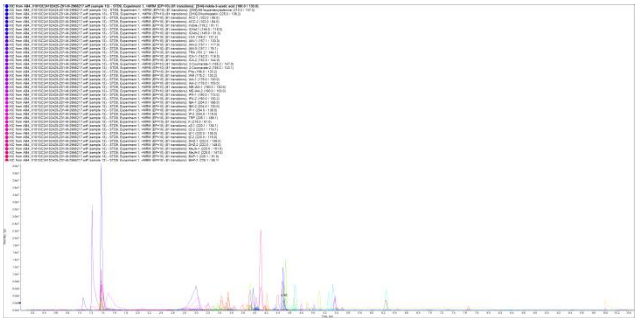

STD10

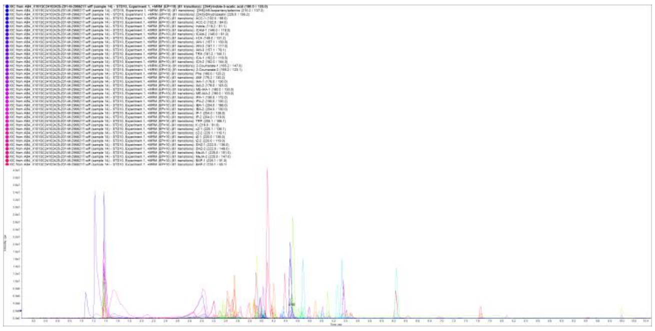

STD11

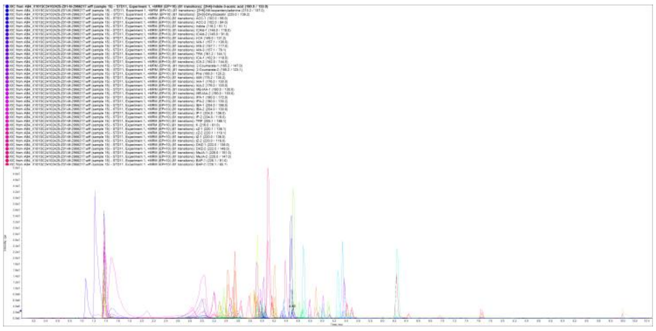

STD12

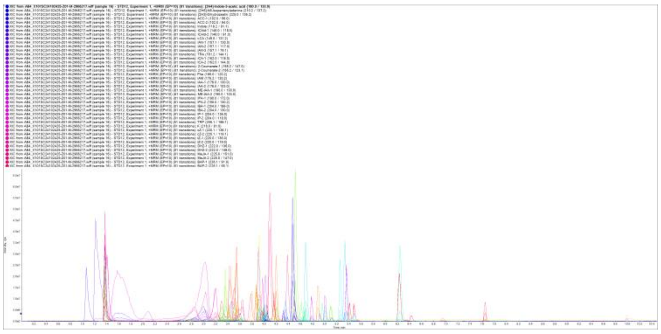

STD13

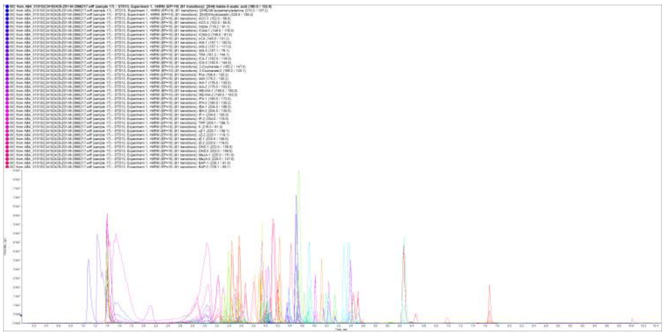

STD14

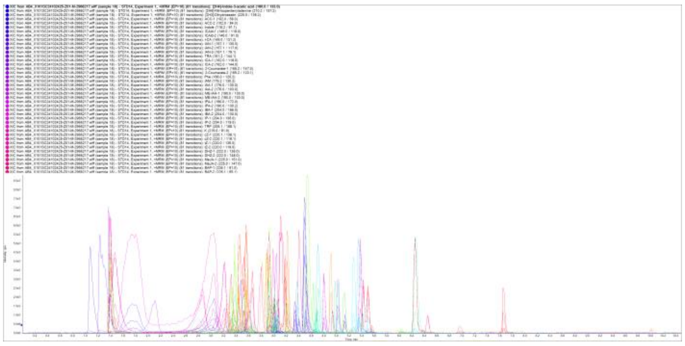

STD15

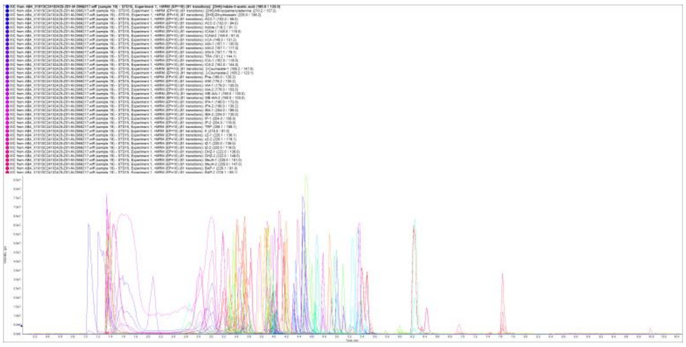

S Fig. 4-2 XIC of samples to be tested (This study)

FZTM250058482-1A

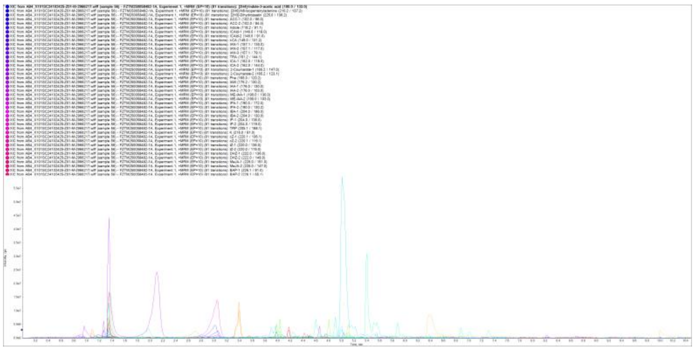

FZTM250058483-1A

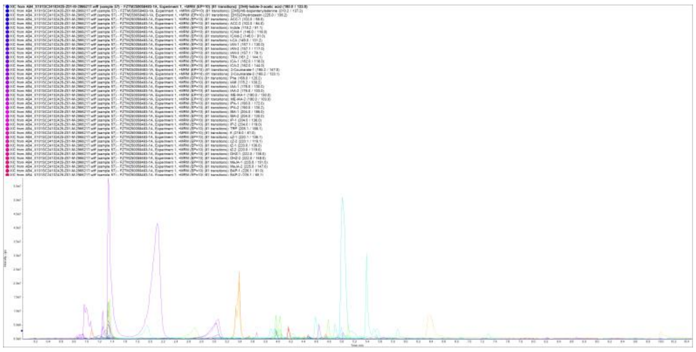

FZTM250058484-1A

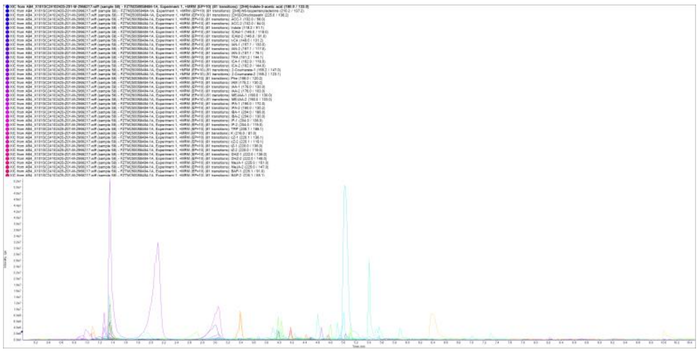

FZTM250058485-1A

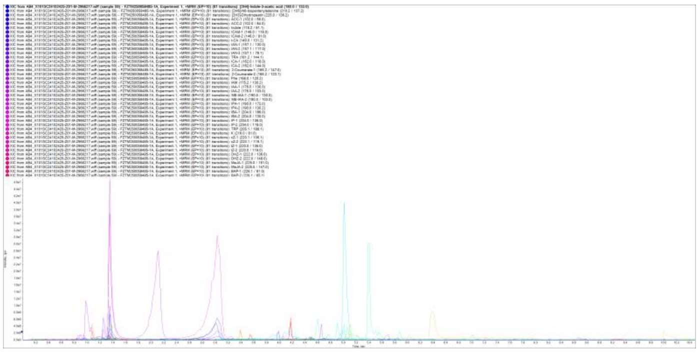

FZTM250058486-1A

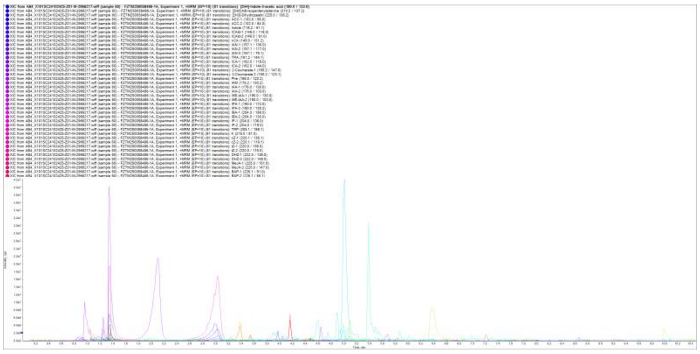

FZTM250058487-1A

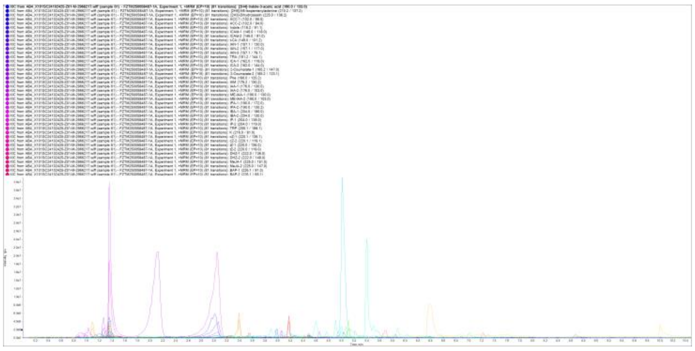

FZTM250058488-1A

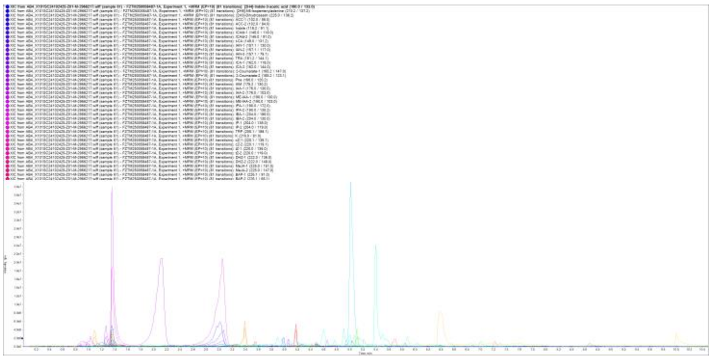

FZTM250058489-1A

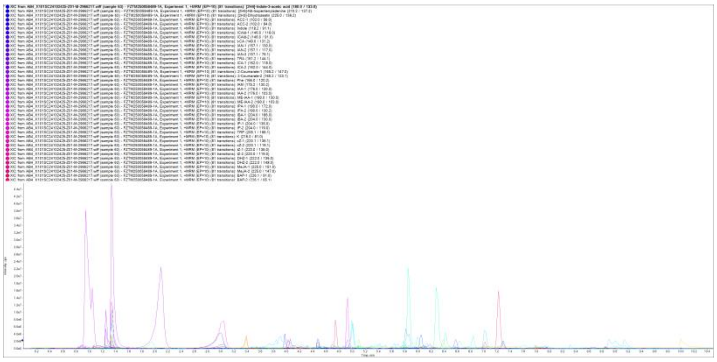

FZTM250058490-1A

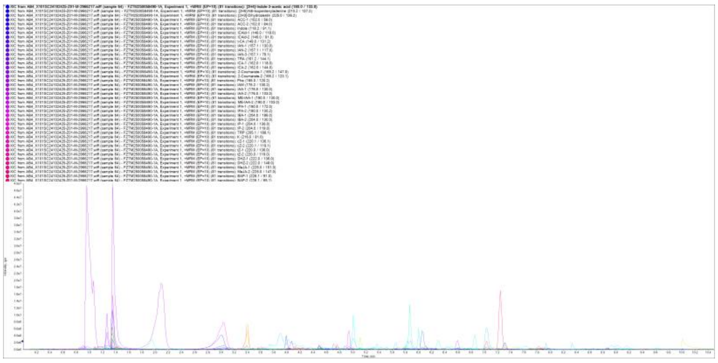

FZTM250058491-1A

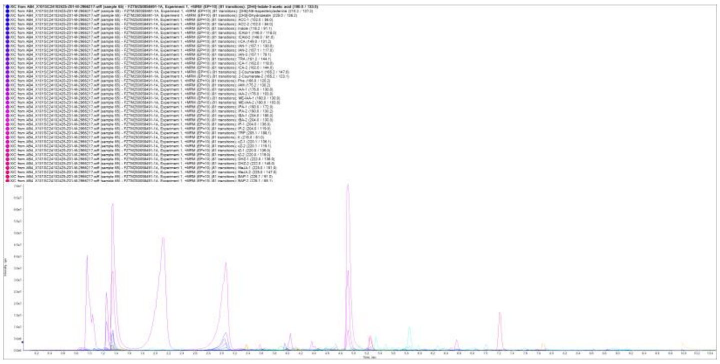

FZTM250058492-1A

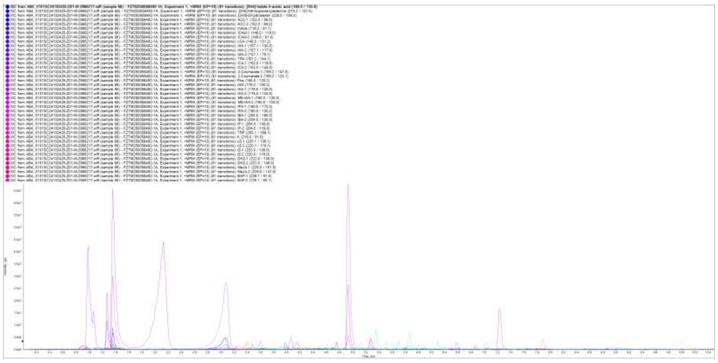

FZTM250058493-1A

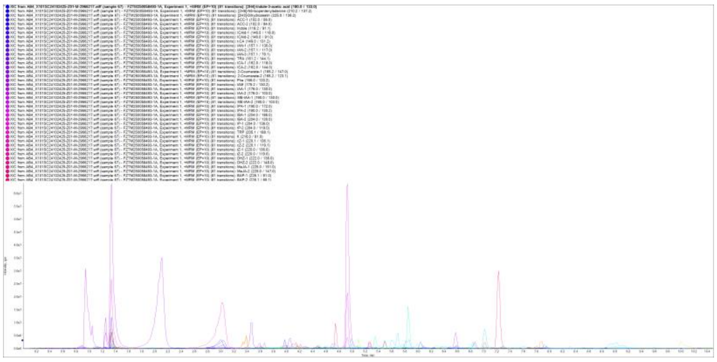

S Fig. 4-3 Calibration curve equation
