# Supplementary material for: Control efficiency and Huanglongbing resistance-related clues mediated by novel Hermetia illucens frass formulation
Source: Front Microbiol. 2026 Aug 4;17:1896630. doi: 10.3389/fmicb.2026.1896630 (PMC13481663; doi:10.3389/fmicb.2026.1896630)
Supplement: Supplementary file 1 [file Data_Sheet_1.zip › S Fig1-5/Fig. 5.pdf]

S Fig. 5 Lipopeptides analysis diagram in HIF by HPLC

The HPLC spectra of the standard curve are cited from Ding et al. (2024).

Surfactin standard sample, Concentration = 500 μm

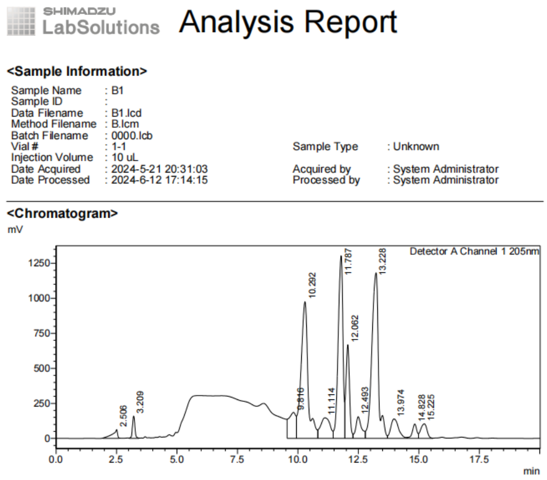

Surfactin standard sample, Concentration = 300 μm

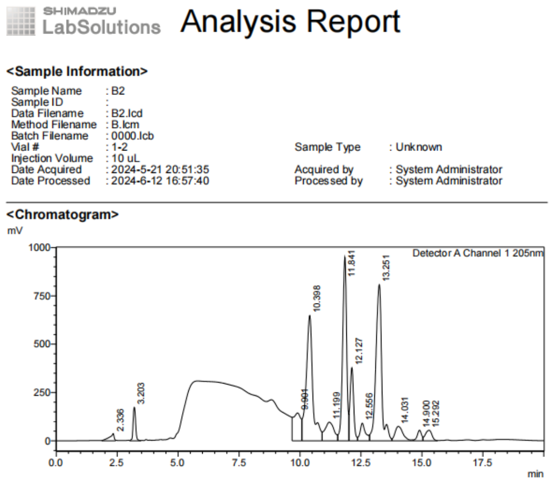

Surfactin standard sample, Concentration = 180 μm

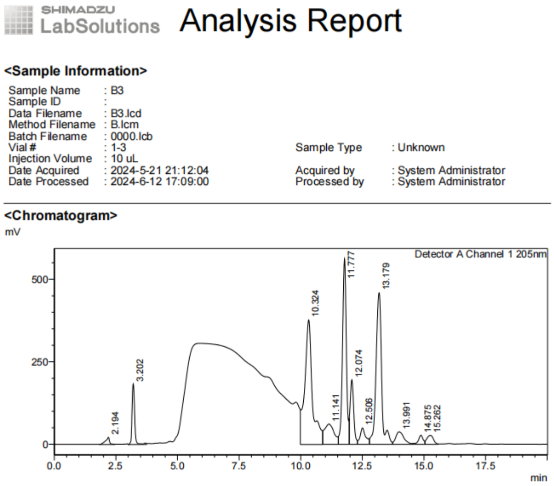

Surfactin standard sample, Concentration = 108 μm

<Sample Information>

Sample Name : B4
Sample ID :
Data Filename : B4.lcd
Method Filename : B.lcm
Batch Filename : 0000.lcb
Vial # : 1-4
Injection Volume : 10 uL
Date Acquired : 2024-5-21 21:32:36
Date Processed : 2024-6-12 17:02:07

Sample Type : Unknown
Acquired by : System Administrator
Processed by : System Administrator

<Chromatogram>

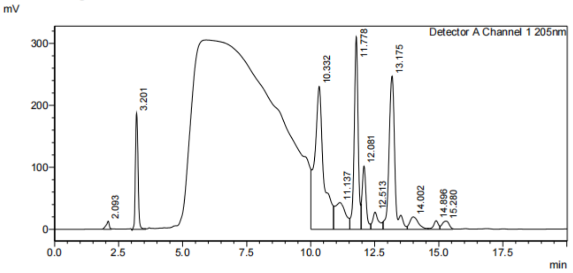

Samples for detection for Surfactin

Samples 1 of HIF （this study）

<Sample Information>

Sample Name : S2
Sample ID :
Data Filename : S2-B001.lcd
Method Filename : B.lcm
Batch Filename : 多个同时跑的程序.lcb
Vial # : 1-2
Injection Volume : 10 uL
Date Acquired : 2024-6-17 02:51:44
Date Processed : 2024-6-17 08:11:53

Sample Type : Unknown
Acquired by : System Administrator
Processed by : System Administrator

<Chromatogram>

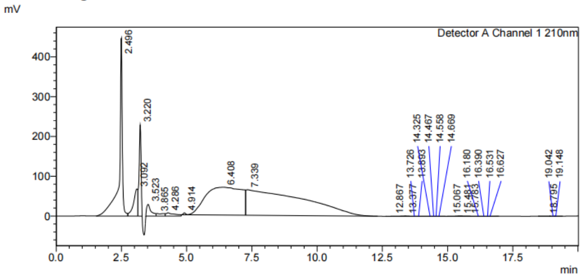

Samples 2 of HIF （this study）

Analysis Report

<Sample Information>

Sample Name : S2
Sample ID :
Data Filename : S2-B.lcd
Method Filename : B.lcm
Batch Filename : 多个同时跑的程序.lcb
Vial # : 1-2
Injection Volume : 10 uL
Date Acquired : 2024-6-16 22:33:25
Date Processed : 2024-6-17 08:03:02

Sample Type : Unknown
Acquired by : System Administrator
Processed by : System Administrator

<Chromatogram>

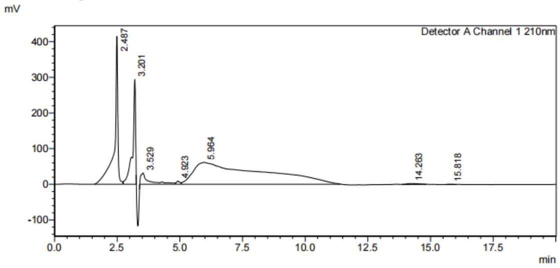

Samples 3 of HIF （this study）

SHIMADZU LabSolutions **Analysis Report**

<Sample Information>

Sample Name	: S3		
Sample ID	:		
Data Filename	: S3-B.lcd		
Method Filename	: B.lcm		
Batch Filename	: 多个同时跑的程序.lcb		
Vial #	: 1-3	Sample Type	: Unknown
Injection Volume	: 10 uL		
Date Acquired	: 2024-6-16 22:53:55	Acquired by	: System Administrator
Date Processed	: 2024-6-17 08:07:20	Processed by	: System Administrator

<Chromatogram>

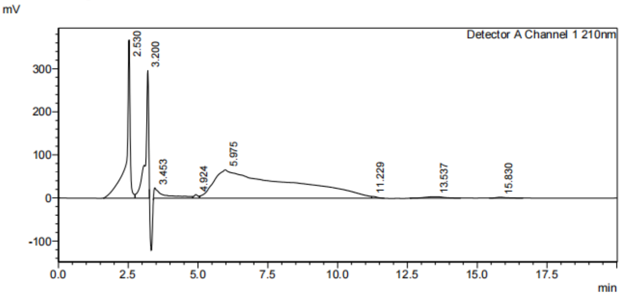

Samples 4 of HIF （this study）

SHIMADZU LabSolutions **Analysis Report**

<Sample Information>

Sample Name	: S3		
Sample ID	:		
Data Filename	: S3-B001.lcd		
Method Filename	: B.lcm		
Batch Filename	: 多个同时跑的程序.lcb		
Vial #	: 1-3	Sample Type	: Unknown
Injection Volume	: 10 uL		
Date Acquired	: 2024-6-17 03:12:13	Acquired by	: System Administrator
Date Processed	: 2024-6-17 03:32:21	Processed by	: System Administrator

<Chromatogram>

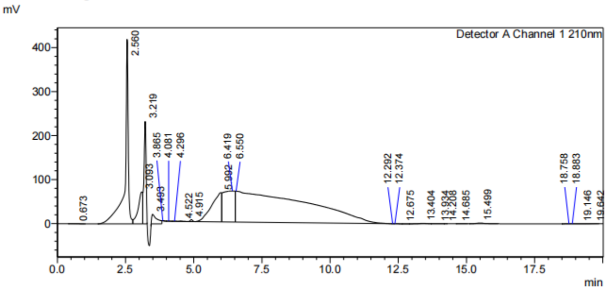

The HPLC spectra of the standard curve are cited from Ding et al. (2024).

Iturin A standard sample, Concentration = 50 μm

SHIMADZU LabSolutions

Analysis Report

<Sample Information>

Sample Name	: Y5		
Sample ID	:		
Data Filename	: Y5.lcd		
Method Filename	: Y.lcm		
Batch Filename	: 1 1 1 .lcb		
Vial #	: 1-12	Sample Type	: Unknown
Injection Volume	: 10 uL		
Date Acquired	: 2024-6-3 18:59:56	Acquired by	: System Administrator
Date Processed	: 2024-6-12 15:50:23	Processed by	: System Administrator

<Chromatogram>

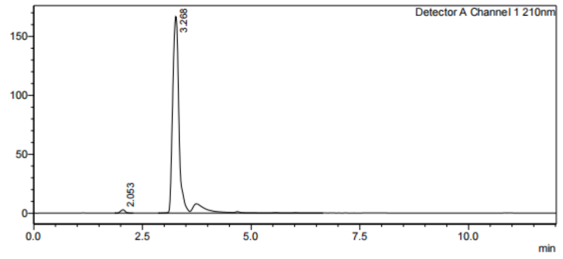

Iturin A standard sample, Concentration = 100 μm

SHIMADZU LabSolutions

Analysis Report

<Sample Information>

Sample Name	: Y4		
Sample ID	:		
Data Filename	: Y4.lcd		
Method Filename	: Y.lcm		
Batch Filename	: 1 1 1 .lcb		
Vial #	: 1-11	Sample Type	: Unknown
Injection Volume	: 10 uL		
Date Acquired	: 2024-6-3 18:47:27	Acquired by	: System Administrator
Date Processed	: 2024-6-12 15:49:45	Processed by	: System Administrator

<Chromatogram>

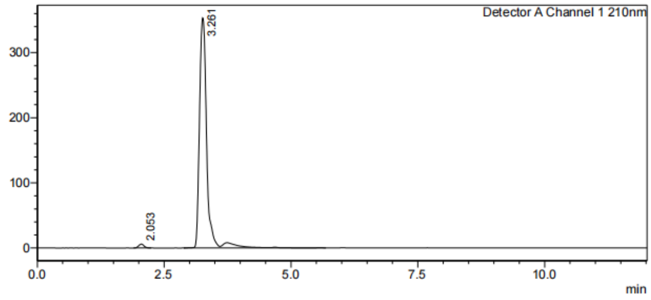

Iturin A standard sample, Concentration = 180 μm

SHIMADZU LabSolutions **Analysis Report**

<Sample Information>

Sample Name	: Y3		
Sample ID	:		
Data Filename	: Y3.lcd		
Method Filename	: Y.lcm		
Batch Filename	: 1 1 1 .lcb		
Vial #	: 1-10	Sample Type	: Unknown
Injection Volume	: 10 uL		
Date Acquired	: 2024-6-3 18:34:57	Acquired by	: System Administrator
Date Processed	: 2024-6-12 15:49:13	Processed by	: System Administrator

<Chromatogram>

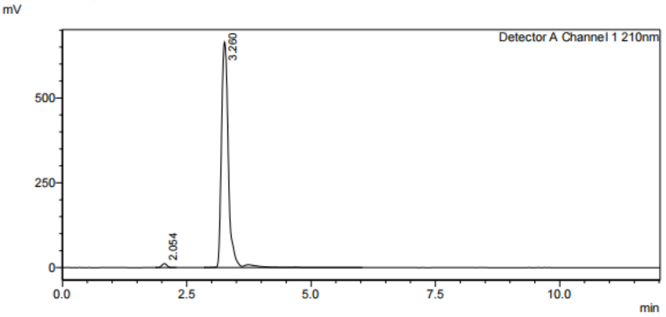

Iturin A standard sample, Concentration = 320 μm

SHIMADZU LabSolutions **Analysis Report**

<Sample Information>

Sample Name	: Y2		
Sample ID	:		
Data Filename	: Y2.lcd		
Method Filename	: Y.lcm		
Batch Filename	: 1 1 1 .lcb		
Vial #	: 1-9	Sample Type	: Unknown
Injection Volume	: 10 uL		
Date Acquired	: 2024-6-3 18:22:25	Acquired by	: System Administrator
Date Processed	: 2024-6-12 15:47:21	Processed by	: System Administrator

<Chromatogram>

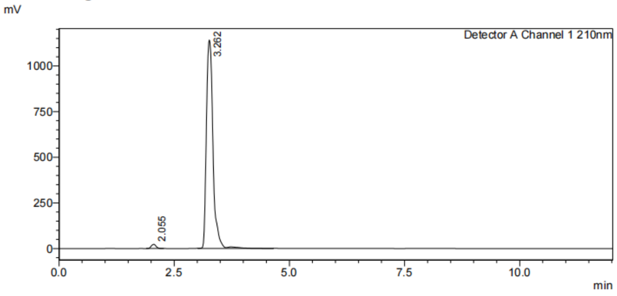

Iturin A standard sample, Concentration = 480 μm

SHIMADZU LabSolutions **Analysis Report**

<Sample Information>

Sample Name	: Y1		
Sample ID	:		
Data Filename	: Y1.lcd		
Method Filename	: Y.lcm		
Batch Filename	: 1 1 1 .lcb		
Vial #	: 1-8	Sample Type	: Unknown
Injection Volume	: 10 uL		
Date Acquired	: 2024-6-3 18:09:55	Acquired by	: System Administrator
Date Processed	: 2024-6-12 16:06:33	Processed by	: System Administrator

<Chromatogram>

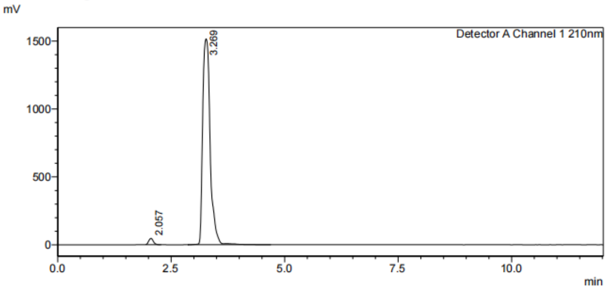

Samples for detection for iturin A

Samples 1 of HIF （this study）

SHIMADZU LabSolutions **Analysis Report**

<Sample Information>

Sample Name : S1
Sample ID :
Data Filename : S1-Y.lcd
Method Filename : Y.lcm
Batch Filename : 多个同时跑的程序.lcb
Vial # : 1-1
Injection Volume : 10 uL
Date Acquired : 2024-6-16 23:14:26
Date Processed : 2024-6-17 01:23:23

Sample Type : Unknown
Acquired by : System Administrator
Processed by : System Administrator

<Chromatogram>

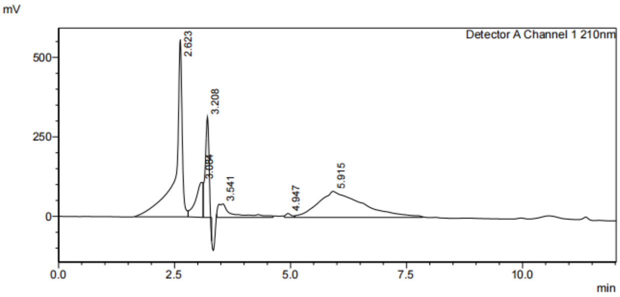

Samples 2 of HIF （this study）

SHIMADZU LabSolutions **Analysis Report**

<Sample Information>

Sample Name : S2
Sample ID :
Data Filename : S2-Y.lcd
Method Filename : Y.lcm
Batch Filename : 多个同时跑的程序.lcb
Vial # : 1-2
Injection Volume : 10 uL
Date Acquired : 2024-6-16 23:26:57
Date Processed : 2024-6-16 23:39:04

Sample Type : Unknown
Acquired by : System Administrator
Processed by : System Administrator

<Chromatogram>

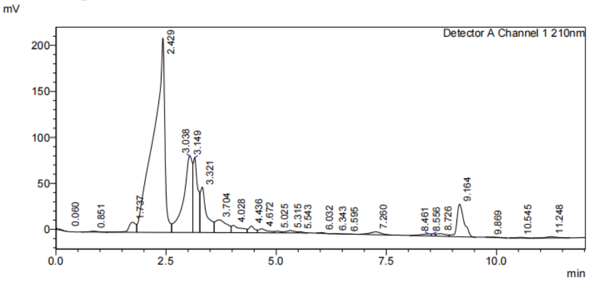

Samples 3 of HIF （this study）

SHIMADZU LabSolutions **Analysis Report**

<Sample Information>

Sample Name : S3
Sample ID :
Data Filename : S3-Y.lcd
Method Filename : Y.lcm
Batch Filename : 多个同时跑的程序.lcb
Vial # : 1-3
Sample Type : Unknown
Injection Volume : 10 uL
Date Acquired : 2024-6-16 23:39:27
Acquired by : System Administrator
Date Processed : 2024-6-16 23:51:35
Processed by : System Administrator

<Chromatogram>

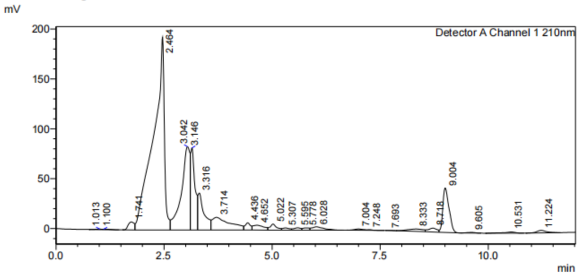

The standard curve equation was show following: (Ding et al., 2024)

Iturin A: y = 36207x – 30246

Surface peak 1: y = 14475x – 553722

Surface peak 2: y = 47409x - 1E+06

Surface peak 3: y = 6802.7x – 304029

Surface Peak 4: y = 4209.1x - 226975
